# Plastome-based backbone phylogeny of East Asian *Phedimus* (Subgenus *Aizoon*: Crassulaceae), with special emphasis on Korean endemics

**DOI:** 10.3389/fpls.2023.1089165

**Published:** 2023-03-14

**Authors:** Yongsung Kim, Seon-Hee Kim, JiYoung Yang, Myong-Suk Cho, Marina Koldaeva, Takuro Ito, Masayuki Maki, Seung-Chul Kim

**Affiliations:** ^1^ Department of Islands and Coast Biodiversity, Division of Botany, Honam National Institute of Biological Resources, Mokpo, Republic of Korea; ^2^ Department of Botany, Graduate School of Science, Kyoto University, Kyoto, Japan; ^3^ Research Institute for Dok-do and Ulleung-do Island, Kyungpook National University, Daegu, Republic of Korea; ^4^ Department of Biological Sciences, Sungkyunkwan University, Suwon, Republic of Korea; ^5^ Botanical Garden-Institute, Far Eastern Branch of the Russian Academy of Sciences, Vladivostok, Russia; ^6^ Botanical Gardens, Tohoku University, Sendai, Japan

**Keywords:** *Aizopsis*, aneuploid series, *Phedimus*, polyploid, subgenus *Aizoon*, plastome, nrDNA ITS

## Abstract

Although the monophyly of *Phedimus* has been strongly demonstrated, the species relationships among approximately 20 species of *Phedimus* have been difficult to determine because of the uniformity of their floral characteristics and extreme variation of their vegetative characters, often accompanied by high polyploid and aneuploid series and diverse habitats. In this study, we assembled 15 complete chloroplast genomes of *Phedimus* species from East Asia and generated a plastome-based backbone phylogeny of the subgenus *Aizoon*. As a proxy for nuclear phylogeny, we reconstructed the nuclear ribosomal DNA internal transcribed spacer (nrDNA ITS) phylogeny independently. The 15 plastomes of subg. *Aizoon* were highly conserved in structure and organization; hence, the complete plastome phylogeny fully resolved the species relationships with strong support. We found that *P. aizoon* and *P. kamtschaticus* were polyphyletic and morphologically distinct or ambiguous species, and they most likely evolved from the two species complex. The crown age of subg. *Aizoon* was estimated to be 27 Ma, suggesting its origin to be in the late Oligocene; however, the major lineages were diversified during the Miocene. The two Korean endemics, *P. takesimensis* and *P. zokuriensis*, were inferred to have originated recently during the Pleistocene, whereas the other endemic, *P. latiovalifolium*, originated in the late Miocene. Several mutation hotspots and seven positively selected chloroplast genes were identified in the subg. *Aizoon*.

## 1 Introduction

The genus *Phedimus* Rafinesque comprises approximately 20 species worldwide and represents a lineage distinct from the more broadly circumscribed “catch-all” genus *Sedum* (Ohba et al., 2000; Mayuzumi and Ohba, 2004). *Phedimus* species are phenotypically like *Sedum* species, but morphological characteristics (i.e., well-developed rhizomes and flattened leaves with dentate margins) and molecular phylogenetic studies strongly support the monophyly of *Phedimus* and its segregation from *Sedum sensu stricto* (Hart, 1995; Ohba et al., 2000; Mayuzumi and Ohba, 2004; Gontcharova et al., 2006; Gontcharova and Gontcharov, 2009). Regarding the phylogenetic position of *Phedimus* within the subfamily Sedoideae, the nrDNA ITS phylogeny suggested that the clade including *Rhodiola* L. and *Pseudosedum* A. Berger is sister to *Phedimus*, while *Sedum sensu stricto* (*Acre* clade) is sister to the *Aeonium* clade (Hart, 1995). In *Phedimus*, two major groups are recognized at the subgeneric rank: subg. *Aizoon* (L.K.A. Koch ex Schönland), Ohba & Turland, and subg. *Phedimus*. Approximately five species of subg. *Phedimus* occur in regions from the Aegean to South Persia and the North Caucasus and have purple or white petals. In contrast, species in subg. *Aizoon*, which has between 12 and 15 species, occurs from East Europe in the South Urals to the Far East and has yellow petals (Grulich, 1984; Gontcharova, 2000; Hart and Bleij, 2003; Gontcharova et al., 2006). Based on the nrDNA ITS phylogeny, two evolutionary lines within the genus *Phedimus* seem to exist: the predominant European lineage *Phedimus* and the Asian lineage *Aizoon*. These two evolutionary lines were further recognized by Chao (2020), but interestingly, two Chinese endemics in subg. *Aizoon*, *P. yangshanicus* (Guangdong Province) and *P. odontophyllus* (W Hubei and SE Sichuan Provinces; Fu et al., 2001), shared their most recent common ancestor with European/Caucasian species rather than with con-subgeneric species of *Aizoon* in East Asia. These results support the idea that the two Chinese endemic species may represent a phylogenetic link between Asian and European *Phedimus* (Chao, 2020).

Of the approximately 15 species in subg. *Aizoon* in East Asia, seven species of *Phedimus* are known to occur in Korea: *P. aizoon*, *P. ellacombeanus*, *P. kamtschaticus*, *P. middendorffianus*, *P. takesimensis*, *P. zokuriensis*, and *P. latiovalifolium*. Of the seven species of *Phedimus* in Korea, four are narrowly restricted to Ulleung Island (*P. takesimensis*), central Korea (*P. zokuriensis* and *P. latiovalifolium*), and southern Korea (*P. ellacombeanus*), whereas *P. aizoon* and *P. kamtschaticus* occur widely throughout the Korean Peninsula. Recently, *P. daeamensis*, which is a narrow endemic to Mt. Daeam, was described in Korea (Choi et al., 2022). Two species that occur widely in Korea, *P. aizoon* and *P. kamtschaticus*, also show a much broader geographical distribution in Russia, China, Mongolia, and Japan. *Phedimus middendorffianus*, which occurs in northern Korea, is also widely distributed in Russia and China. In contrast, some species of the subg. *Aizoon* in East Asia is restricted to certain countries, such as Russia (*P. litoralis* and *P. sichotensis*), Japan (*P. sikokianus*), and China (*P. odontophyllus*, *P. floriferus*, and *P. yangshanicus*). Owing to a lack of synapomorphic characters for species, highly variable morphologies within species, and extensive polyploidy and aneuploidy, the circumscription of species and interspecific relationships within *Phedimus* in East Asia has been problematic (Uhl and Moran, 1972; Chung and Kim, 1989; Amano, 1990; Amano and Ohba, 1992). Individual species within *Phedimus* show diverse morphologies that often intergrade with other recognized taxa, blurring species boundaries and resulting in the recognition of species complexes and polymorphic species with many varieties (Chung and Kim, 1989; Amano, 1990). Although the extent of hybridization in nature is unclear, interspecific relationships within *Phedimus* may be further complicated by hybridization, with documentation of several natural hybrids in the genus (Yoo and Park, 2016).

Nearly half of the species belong to subg. *Aizoon* occur in Korea, three of which are narrow endemics, and phylogenetic relationships among the seven species have been of particular interest (Chung and Kim, 1989; Yoo and Park, 2016; Seo et al., 2020). *Phedimus aizoon* is a polymorphic species with many varieties and is distributed from eastern Siberia to Japan (Amano and Ohba, 1992). In Korea, it has been recognized as a highly variable species, with infraspecific taxa ranging from one (Lee, 1980) to four (Nakai, 1911), five (Lee, 1969), and seven (Oh, 1985). Chung and Kim (1989) showed that *P. aizoon* intergraded with *Phedimus kamtschaticus*, with the observation of several intermediate forms between them. *P. kamtschaticus* is also a highly variable species in Korea in terms of overall status, leaf size, and degree of leaf serration and succulency (Chung and Kim, 1989). Three species present in Korea, *Phedimus ellacombeanus*, *P. takesimensis*, and *P. zokuriensis*, are closely related to *P. kamtschaticus* and are often treated as conspecifics in various taxonomic treatments. *P. ellacombeanus* was originally collected as *P. kamtschaticus* by Maximowicz in 1861 from Hakodate (Hokkaido, Japan) but later described as a new species by Praeger (1917) based on the cultivated materials in Hance’s Herbarium (Chung and Kim, 1989). Although its species status has been controversial, *P. ellacombeanus* has been treated as a distinct species from Korea (Chung and Kim, 1989). This species is also known to occur in the type locality of Hakodate, Hokkaido in Japan, but has been treated as a synonym of *P. aizoon* var. *floribundus* (Nakai) H. Ohba (=*P. kamtschaticus* in Korea) (Ohba, 2002). *Phedimus takesimensis*, a morphologically variable species endemic to Ulleung Island, was first described by Nakai (1919). *Phedimus zokuriensis*, described by Nakai (1939), is endemic to Mt. Sokri and neighboring mountains and is distinguished from congeneric species by weak and creeping stems (Chung and Kim, 1989). It occurs on shaded, wet rocky surfaces in the forests of the mountains of Sokri and Worak in central Korea. Lastly, *P. latiovalifolium* was described recently from Geumdae-bong Peak on Mt. Taebaek (Lee, 1992), and based on morphology, it was suggested to be of hybrid origin between *P. kamtschaticus* and *P. aizoon* or between *P. aizoon* and *P. ellacombeanus* (Lee, 2000; Yoo and Park, 2016).

Although the monophyly of *Phedimus* is strongly supported by molecular phylogenetic studies, interspecific relationships and species entities within subg. *Aizoon* remain poorly understood (Mayuzumi and Ohba, 2004; Gontcharova et al., 2006; Seo et al., 2020). In the eastern Asian Sedoideae phylogeny, Mayuzumi and Ohba (2004) revealed two major lineages within *Phedimus*, that is, subg. *Phedimus* (*P. spurius*) and subg. *Aizoon* (*P. kamtschaticus*, *P. aizoon*, and *P. aizoon* var. *floribundus*, and *P. sikokianus*) had poor resolution in interspecific relationships. Gontcharova et al. (2006) sampled *Phedimus* species primarily from various localities in Primorsky Krai, Russia, and inferred their phylogenetic relationships. In this study, based on nrDNA ITS sequences, they identified two major lineages at the generic rank, *Phedimus* (=subg. *Phedimus*) and *Aizopsis* (=subg. *Aizoon*), with basic chromosome numbers of *x* = 14 and *x* = 16, respectively. In addition, interspecific relationships among primarily Russian Far East species were inferred with limited resolution and relatively low bootstrap support (Gontcharova et al., 2006). To assess the anagenesis of *P. takesimensis* on Ulleung Island, Seo et al. (2020) conducted a phylogenetic analysis based on chloroplast noncoding regions and nrDNA ITS sequences, providing limited support and relationships among Korean populations of five taxa due to low resolution. In addition, a molecular phylogenetic study to trace the cultivar “Tottori Fujita” of *Phedimus* was conducted, finding its origin on Ulleung Island *P. takesimensis* (Han et al., 2020). Lastly, the phylogenetic position of the newly described species from China, *P. yangshanicus*, was assessed based on nrDNA ITS sequences, further confirming species relationships that have previously been identified (Chao, 2020). Therefore, as of today, we have very limited phylogenetic relationships among *Phedimus* species in East Asia, and phylogenomic analysis based on the complete plastome using broader sampling has never been conducted to gain insight into the origin of Korean endemic species. Plastome-based phylogenomic analysis has provided good resolutions and supports, demonstrating its importance in various plant groups (e.g., Xie et al., 2019; Cho et al., 2020; Xie et al., 2020; Yang et al., 2020; Yang et al., 2021). Since the plastome represents the evolutionary history of maternal lineages only, we employed nrDNA ITS sequences to complement the phylogenetic inferences from the plastome sequences. Thus, the aims of this study were to (1) characterize the chloroplast genomes of *Phedimus* species in subg. *Aizoon* in East Asia; (2) generate baseline plastome phylogenetic relationships; (3) infer species relationships based on nrDNA ITS sequences as a proxy for nuclear phylogeny and determine any incongruences between plastome-based and nuclear phylogeny; and (4) gain insights into the origin and evolution of endemic species of *Phedimus* in Korea.

## 2 Materials and methods

### 2.1 Plant materials

**Figure 1:**
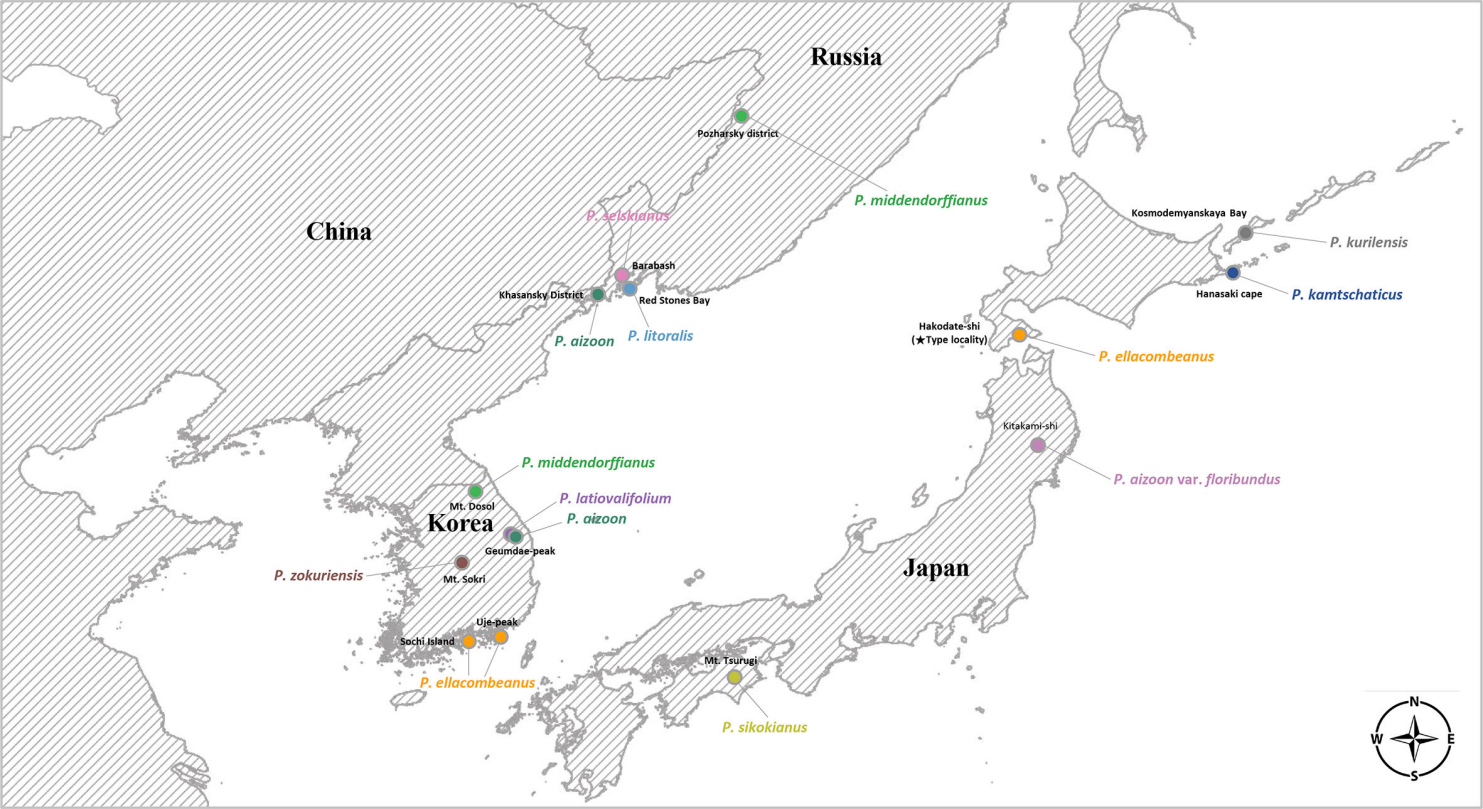
Distribution map of *Phedimus* species sampled in this study.

For each species, we tried to sample at least one accession from its typical geographical range for complete plastome sequencing (**Table 1**, **Figure 1**): *P. sikokianus*, *P. kurilensis*, *P. selskianus*, and *P. litoralis*. In some species (*P. aizoon*, *P. middendorffianus*, *P. ellacombeanus*, and *P. kamtschaticus*), there was more than one accession based on wild or cultivated origins. The plant materials from the Botanical Garden Institute (Vladivostok, Russia) represent transplanted ones from nature into cultivation with accurate species identification. In the case of the Korean endemics, *P. zokuriensis* and *P. latiovalifolium*, we sampled one accession from the type locality, Mt. Sokri, and Geumdae-bong Peak, Mt. Taebaek, respectively, in central Korea. Unfortunately, a newly described Korean endemic, *P. daeamensis*, was not included in this study owing to its recent description and lack of plant materials. Thus, we sequenced the complete plastome of 15 accessions, representing 11 taxa. For sequencing of nrDNA ITS sequences, we sampled a total of 89 accessions representing 10 taxa (**Supplementary Table 1**). All these accessions were of wild origin, with accurate species identification by Seung-Chul Kim, Masayuki Maki, Takuro Ito, and Marina Koldaeva.

**Table 1:** Characteristics of plastomes of the 15 *Phedimus* subg. *Aizoon* accessions used in this study.

Taxon/Locality	GenBank Accession Number	Total cpDNA size (bp)/GC content (%)	LSC size (bp)/GC content (%)	IR size (bp)/GC content (%)	SSC size (bp)/GC content (%)	Number of genes	Number of protein coding genes	Number of tRNA genes	Number of rRNA genes	Number of duplicated genes
1. *Phedimus aizoon* (two accessions)										
Geumdae-bong Peak, Jeongseon-gun, Taebaek-si, Gangwon-do (Korea)	OP344959	151,732/37.7	83,089/35.7	25,983/42.8	16,677/31.9	135	88	37	8	20
Khasansky District, Primorsky Krai (Russia)	OP344958	151,757/37.7	83,104/35.7	25,976/42.8	16,701/31.9	135	88	37	8	20
2. *Phedimus aizoon* var. *floribundus* (one accession)										
Kitakami-shi, Iwate Pref. (Japan)	OP344945	151,748/37.7	83,084/35.7	25,988/42.8	16,688/31.9	135	88	37	8	20
3. *Phedimus ellacombeanus* (three accessions)										
Hakodate-shi, Hokkaido Pref. (Japan)	OP344955	151,869/37.7	83,179/35.7	25,984/42.8	16,722/31.9	135	88	37	8	20
Sochi Island, Namhae-gun, Gyeongsangnam-do (Korea)	OP344957	151,718/37.8	83,055/35.7	25,983/42.8	16,697/32.0	135	88	37	8	20
Uje-bong Peak, Geoje Island, Gyeongsangnam-do (Korea)	OP344956	151,694/37.8	83,034/35.7	25,982/42.8	16,696/31.9	135	88	37	8	20
4. *Phedimus kamtschaticus* (one accession)										
Hanasaki cape, Hanasakiminato, Nemuro-shi, Hokkaido Pref. (Japan)	OP344954	151,849/37.7	83,174/35.7	25,985/42.8	16,705/31.9	135	88	37	8	20
5. *Phedimus kurilensis* (one accession)										
Kosmodemyanskaya Bay, Kunashir Island, Sakhalin Region (Russia)	OP344953	151,845/37.7	83,170/35.7	25,985/42.8	16,705/31.9	135	88	37	8	20
6. *Phedimus latiovalifolium* (one accession)										
Geumdae-bong Peak, Jeongseon-gun, Taebaek-si, Gangwon-do (Korea)	OP344952	151,787/37.7	83,124/35.7	25,982/42.8	16,699/31.9	135	88	37	8	20
7. *Phedimus litoralis* (one accession)										
Red Stones Bay, Reinecke Island, Vladivostok Urban Okrug, Primorsky Krai (Russia)	OP344951	151,761/37.7	83,106/35.7	25,977/42.8	16,701/31.9	135	88	37	8	20
8. *Phedimus middendorffianus* (two accessions)										
Mt. Dosol, Yanggu-eup, Yanggu-gun, Gangwon-do (Korea)	OP344950	151,630/37.8	82,970/35.8	25,982/42.8	16,696/31.9	135	88	37	8	20
Bikin, Pozharsky district, Primorsky Krai (Russia)	OP344949	151,557/37.8	82,890/35.8	25,983/42.8	16,701/31.9	135	88	37	8	20
9. *Phedimus selskianus* (one accession)										
Barabash, Khasansky District, Primorsky Krai (Russia)	OP344948	151,768/37.7	83,112/35.7	25,977/42.8	16,702/31.9	135	88	37	8	20
10. *Phedimus sikokianus* (one accession)										
Mt. Tsurugi, Tokushima Pref. (Japan)	OP344947	151,769/37.7	83,081/35.7	25,985/42.8	16,718/31.7	135	88	37	8	20
11. *Phedimus zokuriensis* (one accession)										
Mt. Sokri, Boeun-gun, Chungcheongbuk-do (Korea)	OP344946	151,746/37.7	83,087/35.7	25,981/42.8	16,697/31.9	135	88	37	8	20

### 2.2 DNA isolation, nrDNA ITS Sanger sequencing, NGS sequencing and plastome assembly/annotation, divergent hotspot identification, and selective pressure analysis

Total genomic DNA was isolated using the DNeasy Plant Mini Kit (Qiagen, Carlsbad, CA, USA), following the manufacturer’s protocol. For nrDNA ITS sequencing, we followed the same protocols of polymerase chain reaction (PCR) amplification and subsequent sequencing as described by Seo et al. (2020). The nrDNA ITS is a multi-copy nuclear gene, and different ribotypes may exist within a single individual, complicating inferences about species relationships. To minimize the potential complications of ribotypes in species relationship inference, we used direct sequencing of PCR products rather than cloning of ribotypes in *Phedimus* species (Gontcharova et al., 2006; Chao, 2020; Seo et al., 2020). In addition, we only included accessions with clean sequencing results. The pairwise sequence divergence was calculated based on the Kimura 2-parameter method (Kimura, 1980) using MEGA11 (Tamura et al., 2021). According to earlier studies, we sequenced the entire plastome (e.g., Kim et al., 2019; Cho et al., 2020; Yang et al., 2021; Yun and Kim, 2022). An Illumina paired-end (PE) genomic library was constructed and sequenced using the Illumina HiSeq platform (Illumina, Inc., San Diego, CA, USA) at Macrogen Corporation (Seoul, Korea). Sequence reads of the plastomes were assembled using the *de novo* genomic assembler Velvet 1.2.10 (Zerbino and Birney, 2008) or NOVOPlasty version 4.3.1 (Dierckxsens et al., 2017). Annotation was performed using Geneious R10 (Biomatters, Auckland, New Zealand) and ARAGORN v1.2.36 (Laslett and Canback, 2004). The pairwise sequence divergence was also calculated based on the Kimura 2-parameter method (Kimura, 1980) using MEGA11 (Tamura et al., 2021). The annotated plastome sequences were deposited in GenBank under accession numbers (**Table 1**). The annotated GenBank (NCBI, Bethesda, MD, USA) format sequence file was used to draw a circular plastid genome map using the OGDRAW software v1.2 (CHLOROBOX) (Lohse et al., 2007). We performed DnaSP v6.10 (Rozas et al., 2017) sliding window analysis, with a step size and window length of 200 bp and 800 bp, respectively, to determine mutation hotspots (that is, the most divergent regions of the plastome). To determine genes under positive selection, a site-specific model was developed using EasyCodeML (Gao et al., 2019) with CodeML algorithms (Yang, 1997). Seven codon substitution models (M0, M1a, M2a, M3, Mt, M8, and M8a) were constructed and compared to detect positively selected sites using the likelihood ratio test (LRT).

### 2.3 Phylogenetic analysis

For the ITS phylogeny, we included 106 accessions, representing two species (three accessions) of subg. *Phedimus* (*S. obtusifolius* was not available) and 15 species (103 accessions) of subg. *Aizoon*, and based on previous studies, the genus *Rhodiola* was used as an outgroup (Mayuzumi and Ohba, 2004; Gontcharova et al., 2006; Chao, 2020; Messerschmid et al., 2020) (**Supplementary Table 1**). Of the 106 accessions, newly sequenced ITS sequences (a total of 89 accessions) included *P. latiovalifolium* (16 accessions), *P. zokuriensis* (four accessions), *P. takesimensis* (14 accessions), *P. middendorffianus* (six accessions), *P. kurilensis* (one accession), *P. ellacombeanus* (nine accessions from Korea and Japan), *P. litoralis* (two accessions), *P. aizoon* (18 accessions), *P. selskianus* (one accession), and *P. kamtschaticus* (18 accessions). All the sequences were edited and assembled using Sequencher v4.2.2 (Gene Codes, Ann Arbor, MI, USA) and Geneious R10 (Biomatters, Auckland, New Zealand). The ITS sequences for the following species (*P. stellatus*, *P. spurius*, *P. odontophyllus*, *P. yangshanicus*, *P. hybridus*, *P. sichotensis*, and *P. sikokianus*) were also obtained from GenBank. We included these species (totaling 17 accessions) because some represent members of subg. *Phedimus* (*P. stellatus*, *P. spurius*, *P. odontophyllus*, and *P. yangshanicus*), and other species (*P. hybridus*, *P. selskianus*, *P. sichotensis*, and *P. sikokianus*) have distinct species diagnostic features, minimizing potential misidentifications and providing overall species relationships in subg. *Aizoon*. The sequences were aligned using Clustal X v1.83 (Thompson, 1997) with a final manual adjustment using MacClade (Maddison and Maddison, 2002). Maximum likelihood (ML) analysis was conducted using IQ-TREE v1.4.2 (Nguyen et al., 2015), with 1,000 replicate bootstrap (BS) analyses, based on the best-fit model of TIM3e + G4 selected by ModelFinder (Kalyaanamoorthy et al., 2017). For plastome phylogeny, the complete plastome sequences were aligned using MAFFT v7 (Katoh and Standley, 2013), and an ML phylogenetic tree was constructed using IQ-TREE with 1,000 bootstrap replicates (Nguyen et al., 2015). The best-fit evolutionary model for the complete plastome sequences, TVM + F + I + G4, was selected based on ModelFinder (Kalyaanamoorthy et al., 2017), implemented in IQ-TREE v1.4.2. A representative species of *Rhodiola* was used as the outgroup in ITS analysis (Messerschmid et al., 2020). Given the lack of representative plastomes of subg. *Phedimus*, we included *Rhodiola* species as part of the ingroup and *Umbilicus* as the outgroup.

### 2.4 Molecular dating

Divergence times based on complete plastome sequences were estimated using the Bayesian method (Drummond et al., 2006) using BEAST version 1.10.4 (Suchard et al., 2018). The XML file for analysis was prepared using the Bayesian evolutionary analysis utility (BEAUTi). Owing to the lack of reliable fossils of *Phedimus* and related genera of Crassulaceae, we considered two secondary calibration points based on ITS phylogeny: an estimated *Rhodiola* crown mean age of 7.17 Myr and a standard deviation of 4.87, giving a range of 3.09–12.03 Myr, and the *Phedimus* and *Rhodiola* clade stem mean age of 39.43 Myr, and a standard deviation of 14.31, giving a range of 24.91–53.74 Myr (Messerschmid et al., 2020). We used the Yule process speciation prior, a lognormal relaxed clock model, and the GTR-γ substitution model, and then the ucld.mean parameter was specified to be uniform with 0.333 as the initial value, 0.00 as the lower limit, and 1 as the upper limit (Drummond et al., 2006). Posterior distributions for each parameter were estimated using an MCMC run for 400 million generations with a sampling frequency of 50,000 generations. The posterior distribution of all statistics was checked using Tracer version 1.5 (Rambaut and Drummond, 2009) to assess convergence and confirm whether the effective sample sizes (ESS) for all parameters were larger than 200 (Drummond et al., 2012). In addition, we used TreeAnnotator version 1.5 (http://beast.bio.ed.ac.uk/TreeAnnotator) to produce a maximum credibility tree of mean divergence time and 95% highest posterior density (HPD) intervals with a posterior probability (PP) limit (0.5) after removing the first 25% of trees as burn-in (Drummond et al., 2012).

## 3 Results

### 3.1 Characterization of chloroplast genomes, mutation hotspots, and positively selected genes in *Phedimus* species of subg. *Aizoon*

**Figure 2:**
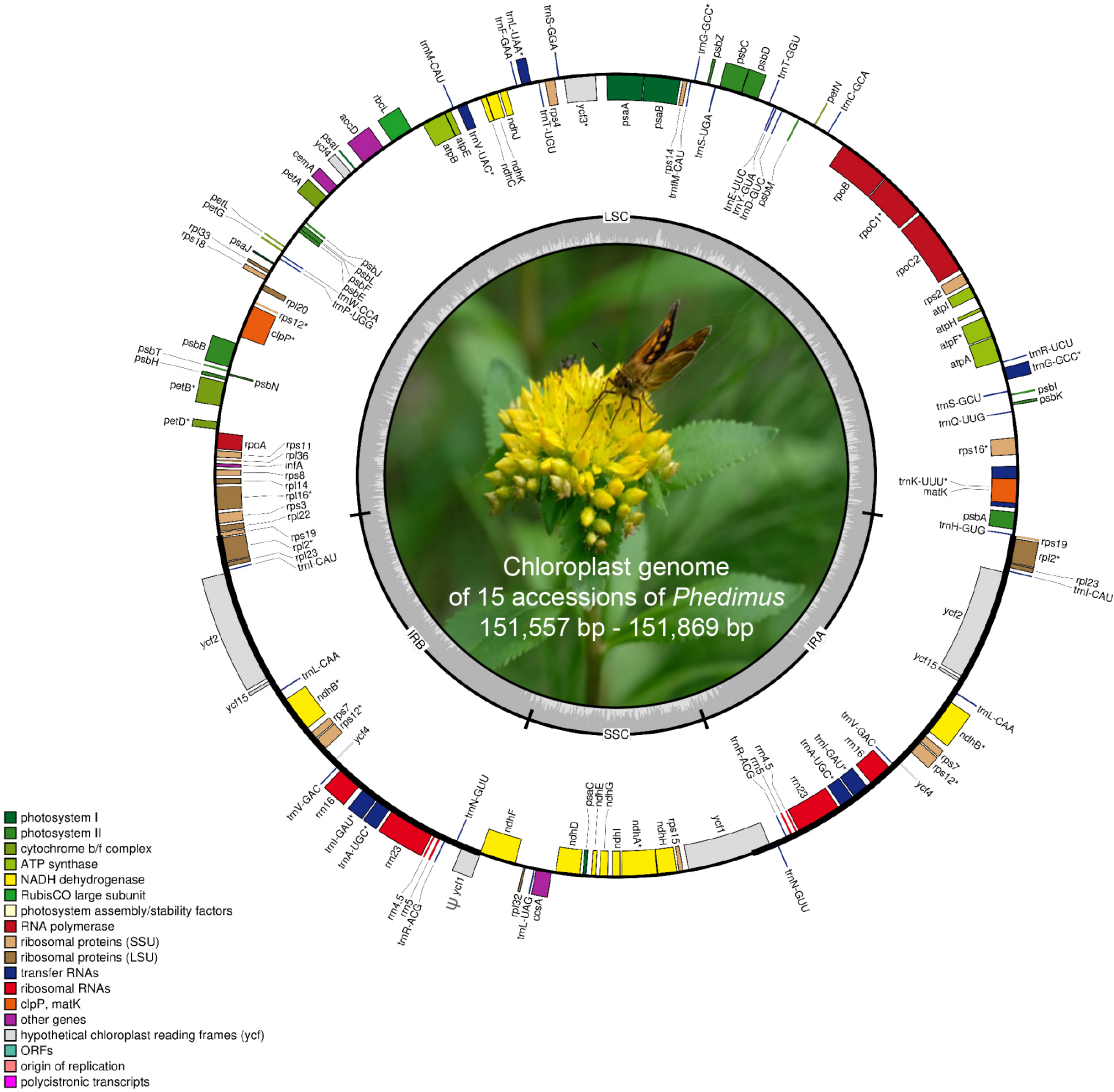
Complete plastome map of *Phedimus* subg. *Aizoon* species in East Asia. The genes inside and outside of the circle are transcribed in the clockwise and counterclockwise directions, respectively. Genes belonging to different functional groups are shown in different colors. The thick lines indicate the extent of the inverted repeats (IR_A_ and IR_B_) that separate the genomes into small single copy (SSC) and large single copy (LSC) regions.

**Figure 3:**
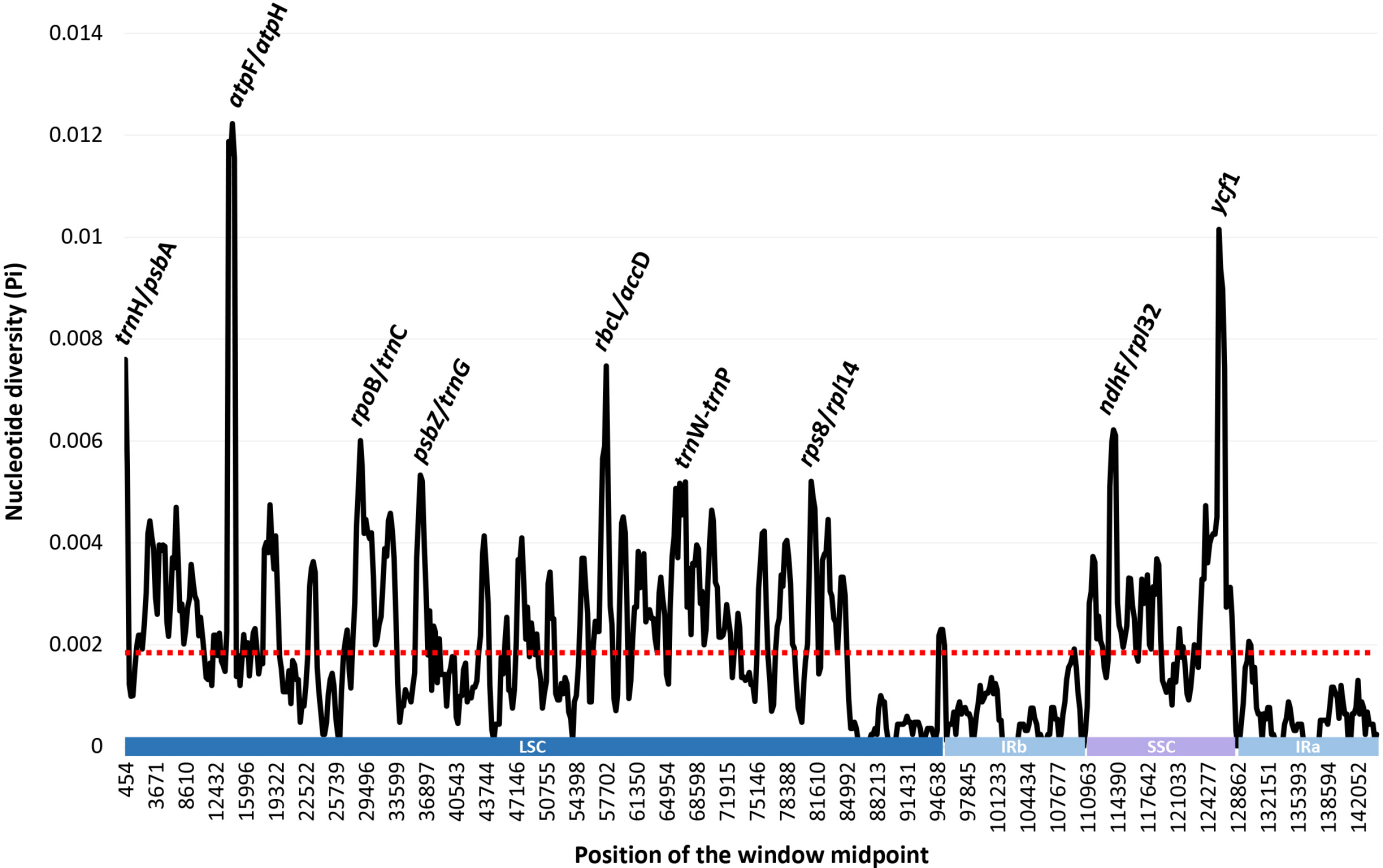
Sliding window analysis of the whole plastomes of 15 accessions (11 taxa) of *Phedimus* subg. *Aizoon*.

The complete plastome length of the subg. *Aizoon* ranged from 151,557 bp (*P. middendorffianus;* Primorsky Krai, Russia) to 151,869 bp (*P. ellacombeanus*; Hokkaido, Japan) (**Table 1**; **Figure 2**). The large single copy (LSC) region, small single copy (SSC) region, and two inverted repeat (IR) regions ranged from 82,890 bp (*P. middendorffianus*; Primorsky Krai, Russia) to 83,179 bp (*P. ellacombeanus*; Hokkaido, Japan), 16,677 bp (*P. aizoon*; Geumdae-bong Peak, Korea) to 16,718 bp (*P. sikokianus*; Tokushima Pref., Japan), and 25,976 bp (*P. aizoon*; Primorsky Krai, Russia) to 25,988 bp (*P. aizoon* var. *floribundus*; Iwate Pref. Japan), respectively (**Table 1**). All 15 accessions of the subg. *Aizoon* contains 135 genes, including 88 protein-coding, eight ribosomal RNA, and 37 transfer RNA genes. The overall guanine-cytosine (GC) content ranged from 37.7% to 37.8%, and 20 duplicate genes were found in the IR regions. Sliding window analysis using the DnaSP program identified several highly variable genic and intergenic (“–”) regions in 15 plastomes of subg. *Aizoon*: *atpF*–*atpH* (Pi = 0.01223), *ycf1* (Pi = 0.01015), *trnH*–*psbA* (Pi = 0.0076), *rbcL*–*accD* (Pi = 0.00747), *ndhF*–*rpl32* (Pi = 0.00622), *rpoB*–*trnC* (Pi = 0.00601), *psbZ*–*trnG* (Pi = 0.00533), *trnW*–*trnP* (Pi = 0.0052), and *rps8*–*rpl14* (Pi = 0.00489) (**Figure 3**). The average nucleotide diversity value (Pi) over the entire plastome was 0.00188. Among the conserved genes, we identified seven genes with positively selected sites (**Table 2**). These genes included the c-type cytochrome synthesis gene (*ccsA*), chloroplast envelope membrane protein (*cemA*), maturase K gene (*matK*), NADH dehydrogenase subunit gene (*ndhC*), photosystem II protein gene (*psbJ*), cytoplasmic ribosomal protein L22 gene (*rpl22*), and RNA polymerase C2 gene (*rpoC2*).

**Table 2:** Positively selected genes and sites detected in the plastomes of *Phedimus* subg. *Aizoon* species.

Gene name	Models	np	ln L	Likelihood Ratio Test *p*-Value	Positively Selected Sites
*ccs*A	M8	45	−1,393.266207	0.000000000	F 0.998**
	M7	43	−1,465.917908		
*cem*A	M8	45	−1,031.895178	0.000000000	105 H 0.950*
	M7	43	−1,101.793428		
*mat*K	M8	45	−2,263.614460	0.000000000	199 L 0.971*
	M7	43	−2,339.739355		
*ndh*C	M8	45	−527.638862	0.000000000	26 L 0.976*
	M7	43	−596.432768		
*psb*J	M8	45	−165.418110	0.000013861	27 I 0.974*; 28 G 0.952*; 29 L 1.000**; 30 G 0.995**; 32 S 0.955*; 33 L 0.993**
	M7	43	−176.604574		
*rpl*22	M8	45	−619.287439	0.000013861	18 S 0.972*,55 F 0.974*
	M7	43	−675.381579		
*rpo*C2	M8	45	−6,031.704371	0.014323525	1375 F 0.979*
	M7	43	−6,035.950223		

*p <0.05; **p <0.01. np represents the degrees of freedom.

### 3.2 Complete plastome sequence phylogeny

**Figure 4:**
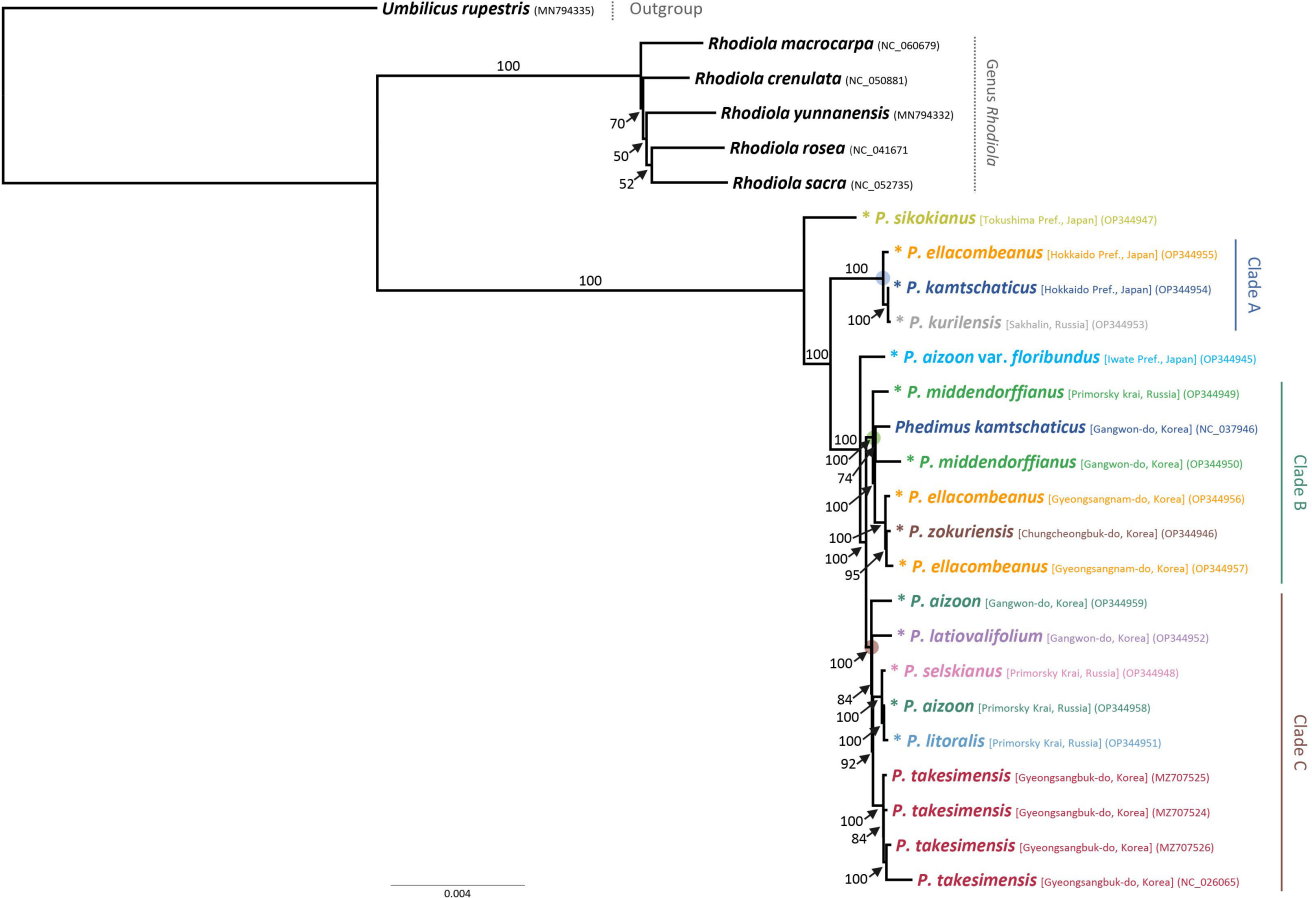
Maximum likelihood phylogeny of subg. *Aizoon*, including 11 species of *Phedimus*, based on complete plastome sequences. Bootstrap support values >50% are shown above and below branches. Asterisked 15 accessions are newly obtained in this study.

For the first time, we obtained robust and well-resolved whole plastome-based phylogenetic relationships among representative species of subg. *Aizoon* in East Asia (**Figure 4**). The plastome tree suggested that *P. sikokianus*, which is diploid and endemic to southern Japan, diverged early within the subg. *Aizoon* and further identified three major lineages, clades A–C. The first lineage, clade A (100% bootstrap support, BS), included *P. ellacombeanus*, sampled as *P. aizoon* var. *floribundus* from the type locality of the species in Japan (Hokkaido, Japan), *P. kurilensis* (Sakhalin, Russia), and *P. kamtschaticus* (Hokkaido, Japan). All these taxa occur from northern Japan (Hokkaido) to the southern Kuriles (Russia). In this clade, *P. kurilensis*, endemic to the southern Kuril Islands and considered a synonym of *P. sikokianus*, is sister to *P. kamtschaticus* from Hokkaido, Japan (100% BS). After the divergence of clade A, *P. aizoon* var. *floribundus* (Iwate, Japan) is sister to the remaining clades of B and C (100% BS). The second lineage, clade B (100% BS), included *P. middendorffianus* (two accessions from Russia and Korea), *P. kamtschaticus* (Korea), two accessions of *P. ellacombeanus*, and *P. zokuriensis* (Korea). One Korean accession of *P. middendorffianus* sampled from Gangwon-do Province is sister to *P. kamtschaticus*, which was also sampled from Gangwon-do Province (moderate support, 74% BS). The central South Korean Peninsula Endemic *P. zokuriensis* is sister to the *P. ellacombeanus* sampled from Sochi Island, which is in the southeastern part of the Korean Peninsula (95% BS). Lastly, the third lineage, clade C (100% BS), included two Korean endemic species (*P. latiovalifolium* and *P. takesimensis*), *P. aizoon* (Korea), and species from Russia (*P. selskianus*, *P. litoralis*, and *P. aizoon*). The monophyletic Ulleung Island endemic *P. takesimensis* shares its most recent common ancestor with *Phedimus* species from Russia (*P. selskianus*, *P. litoralis*, and *P. aizoon*; 92% BS). The central South Korean peninsula endemic *P. latiovalifolium* is sister to the clade containing species from Russia and the Ulleung Island endemic *P. takesimensis* (84% BS). Overall, plastome-based phylogeny of subg. *Aizoon* suggested that *P. takesimensis* is monophyletic, while the more widely distributed *P. aizoon* and *P. kamtschaticus* appear to be polyphyletic, which requires further confirmation based on multiple accessions. Pairwise sequence divergence based on Kimura 2-parameter distances was shown in **Supplementary Table 2**. The average pairwise sequence divergence for all 15 accessions was 0.189%, ranging from 0.008% (between *P. kamtschaticus* OP344954 and *P. kurilensis* OP344953) to 0.425% (between *P. takesimensis* NC026025 and *P. sikokianus* OP344947). The average pairwise sequence divergence for four accessions of *P. takesimensis* on Ulleung Island and three accessions of *P. ellacombeanus* was 0.053% and 0.215%, respectively. The type locality accession of *P. ellacombeanus* (OP344955) in Japan showed quite divergent pairwise sequence divergence from its conspecific populations sampled in Korea: 0.307% between type locality accession OP344955 and two Korean accessions versus 0.030% between two Korean accessions of *P. ellacombeanus*.

### 3.3 nrDNA ITS phylogeny

**Figure 5:**
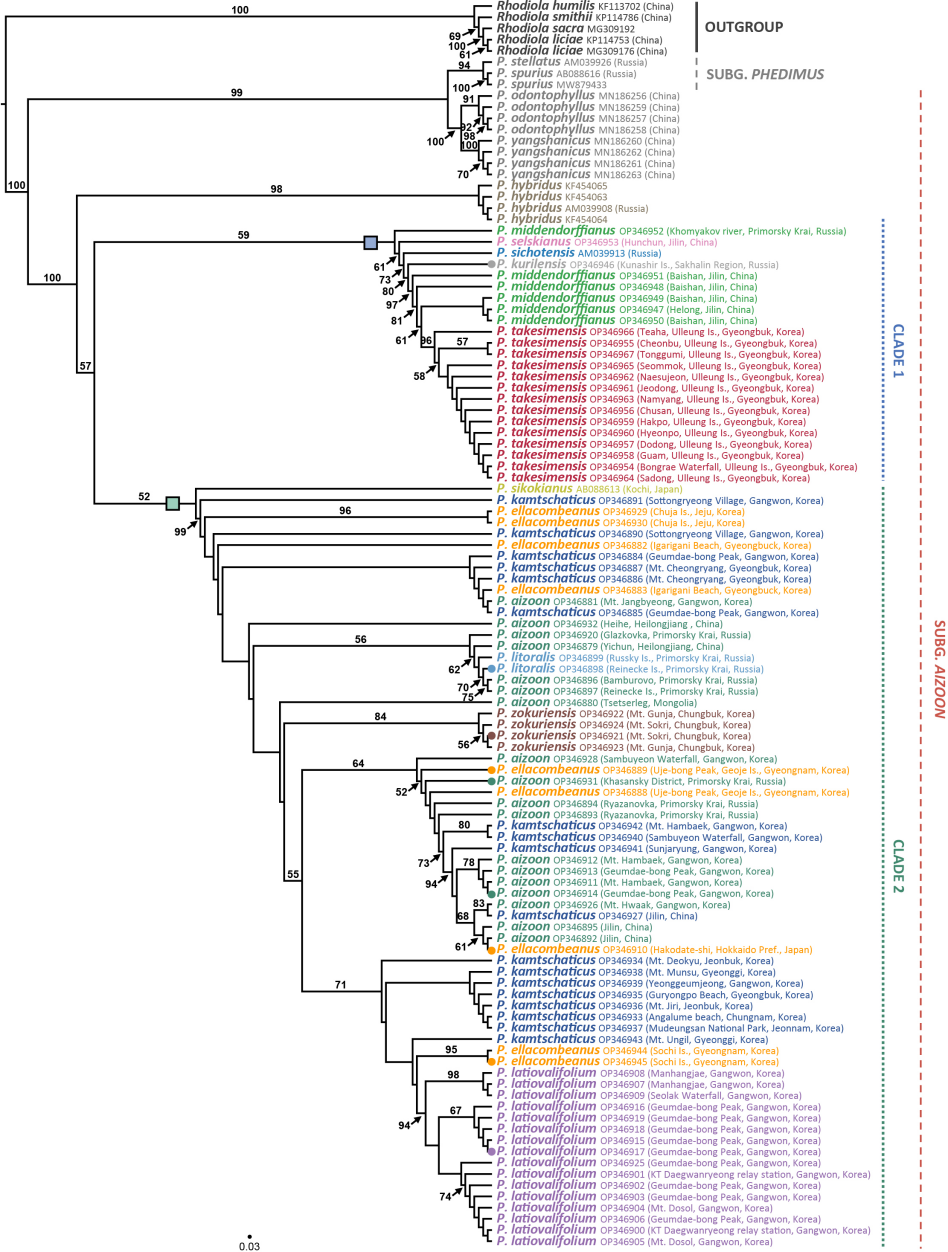
Maximum likelihood phylogeny of the genus *Phedimus* based on the nrDNA ITS sequences. Bootstrap support values >50% are shown below branches. Accessions in circles are sequenced for their complete plastomes, and the rectangles represent two major clades of subg. *Aizoon*.

The ITS ML tree revealed two major lineages within the genus *Phedimus* (**Figure 5**; phylogram shown in **Supplementary Figure 1**). Pairwise sequence divergence based on the Kimura 2-parameter distances was shown in **Supplementary Table 3**. For all 106 accessions (including two subgenera *Phedimus* and *Aizoon*), the average pairwise sequence divergence was 1.654%: 2.967% for species of subg. *Phedimus* and 1.371% for species of subg. *Aizoon*. Pairwise sequence divergence between two species of subg. *Aizoon* (i.e., *P. odontophyllus* and *P. yangshanicus*) and the remaining species was 7.064%, while between *P. odontophyllu*s/*P. yangshanicus* and subg. *Phedimus* was 1.254%. Accordingly, *P. odontophyllus* and *P. yangshanicus* are more closely related to species of subg. *Phedimus* than to consubgeneric species in subg. *Aizoon*. The first lineage (99% BS) included two species of subg. *Phedimus* (*P. stellatus* and *P. spurius*) and two species of subg. *Aizoon* (*P. odontophyllus* and *P. yangshanicus*), confirming non-monophyly of subg. *Aizoon* (Chao, 2020). According to ITS sequence divergence, two species of subg. *Aizoon*, *P. odontophyllus* and *P. yangshanicus*, are more closely related to members of subg. *Phedimus* than those of subg. *Aizoon* (**Supplementary Table 3**). The second lineage (100% BS) included all but two species of subg. *Aizoon* (100% BS), and monophyletic *P. hybridus* (98% BS) diverged first within this clade. Although bootstrap support values within the subg. *Aizoon* restricted us from rigorously inferring species monophyly and interspecific relationships, but some species relationships could be postulated with caution. The ITS tree identified one weakly supported clade 1 (59% BS), which included *P. middendorffianus*, *P. selskianus*, *P. kurilensis*, *P. sichotensis*, and *P. takesimensis*. The Ulleung Island endemic *P. takesimensis* is monophyletic (96% BS) and embedded within the non-monophyletic *P. middendorffianus*, sampled primarily from northeastern (Jilin) China. One accession of *P. middendorffianus* sampled from Primorsky Krai, Russia, was sister to the remaining accessions within this clade. *Phedimus sichotensis* (AM039913 from GenBank), which is often treated as a subspecies of *P. middendorffianus* (*P. middendorffianus* subsp. *sichotensis*), is sister to the clade containing primarily *P. middendorffianus* and *P. takesimensis* (73% BS). The southern Kuriles (Russia) endemic *P. kurilensis* accession sequenced in this study is sister to the clade containing *P. middendorffianus* and *P. takesimensis* (80% BS).

In addition, the ITS tree also suggested that *P. sikokianus* (AB08863 from GenBank), which is narrowly endemic to southern Japan (Shikoku), is sister to the members of clade 2 (52% BS weak support; thus, it could be either sister to clade 1 or 2). Excluding *P. sikokianus*, the remaining clade 2 is strongly supported (99% BS). Within clade 2, the central South Korean Peninsula endemics *P. zokuriensis* and *P. latiovalifolium* are monophyletic (84% and 94% BS, respectively), whereas the widely distributed *P. aizoon* and *P. kamtschaticus* are not monophyletic. The ITS tree also showed that *P. litoralis*, endemic to Russia, was deeply embedded within the *P. aizoon* lineage sampled primarily from Primorsky Krai (Russia) and Heilongjiang (China) (56% BS). *Phedimus ellacombeanus* sampled from the type locality of Japan and Korea was polyphyletic. One accession sampled from southeastern Korea, Igarigani Beach, Pohang (Gyeongsangbuk-do Province), is closely related to *P. kamtschaticus*, sampled from various parts of the Korean Peninsula, and *P. aizoon*, sampled from Gangwon-do Province, Korea. Conversely, two accessions sampled from southeastern Korea, that is, Uje-bong Peak and Geoje Island (Gyeongsannam-do Province), are closely related to *P. aizoon* and *P. kamtschaticus* collected from Korea, Russia, and China. Furthermore, two accessions sampled from Hachuja Island in the southern part of Korea are sister to the clade containing all but *P. sikokianus* and one accession of *P. kamtschaticus* (Korea) in clade 2. Two accessions of *P. ellacombeanus* from Sochi Island in the southern part of Korea share their most recent common ancestor with *P. latiovalifolium* in Gangwon-do Province. Lastly, one accession collected from the type locality of *P. ellacombeanus* in Hakodate (southern Hokkaido, Japan) is embedded within *P. aizoon* from northeastern (Jilin) China (61% BS).

Several distinct lineages of highly polyphyletic *P. aizoon* were found exclusively in clade 2 (**Figure 5**), including a lineage largely from Gangwon-do Province (Korea)/Jilin (China) and a lineage primarily from Primorsky Krai (Russia), Mongolia, and *P. litoralis* (56% BS). Several lineages of *P. kamtschaticus* were also revealed in Korea, including one lineage sampled from various parts of Korea (<50% BS) and the other lineage, which is sister to the clade of *P. ellacombeanus* (Sochi Island) and *P. latiovalifolium*. Both *P. aizoon* and *P. kamtschaticus* were not only intermixed with each other but also closely related to other congeneric species, such as *P. ellacombeanus* and *P. litoralis*.

### 3.4 Molecular dating

**Figure 6:**
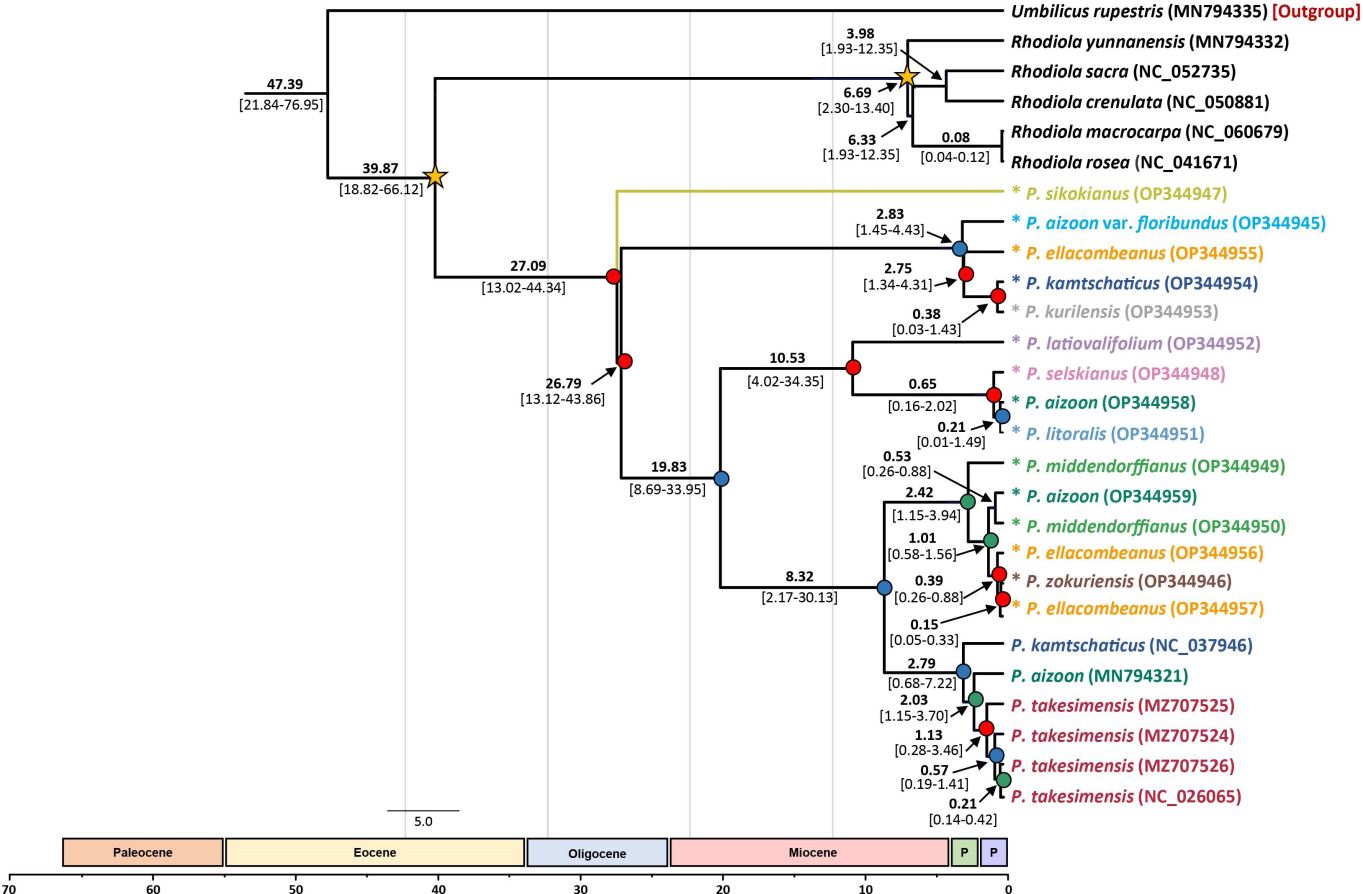
Dated chronogram showing divergence time of 21 accessions of subg. *Aizoon* plastomes. Estimated mean ages are shown for each node, with 95% high posterior density (HPD) in brackets. Internal node posterior probability (PP) is shown in red (PP ≥0.95), blue (0.95> PP >0.75), and green (0.75 >PP). Two calibration points are shown as asterisked nodes based on a previous study (Messerschmid et al., 2020).

Based on the complete plastome sequences, we estimated the divergence times of the major lineages within the subg. *Aizoon* (**Figure 6**). Without any representative species from subg. *Phedimus*, the age of subg. *Aizoon* was estimated to be 27.09 Ma (95% HPD, 13.02–44.34 Ma), suggesting its origin in the late Oligocene. Within the subg. *Aizoon*, the early divergence of diploid *P. sikokianus* in southern Japan was immediately followed by the divergence of the remaining lineages (26.79 Ma; 95% HPD, 13.12–43.86 Ma). The age of the estimated divergence of the Ulleung Island endemic *P. takesimensis* from its continental sister species was estimated to be 2.03 Ma (95% HPD, 1.15–3.70 Ma), suggesting its early colonization soon after the formation of Ulleung Island (ca. 1.8 Ma). The estimated divergence of the narrow endemic *P. zokuriensis* in Korea from its sister *P. ellacombianus* was inferred to be very recent at 0.15 Ma (95% HPD, 0.05–0.33 Ma) during the Pleistocene (late Ionian), whereas that of the other narrow Korean endemic *P. latiovalifolium* was inferred at 10.53 Ma (95% HPD, 4.02–34.35 Ma) during the late Miocene (late Messinian).

## 4 Discussion

### 4.1 Plastome conservation, mutation hotspots, and positively selected genes in subg. *Aizoon*

Genome size, gene order, and number were highly conserved within the subg. *Aizoon* (**Table 1**). In addition, the overall genome size, gene numbers, and GC content were like those of the sister lineage *Rhodiola* and other closely related genera (*Hylotelephium*, *Kalanchoe*, *Orostachys*, *Rosularia*, *Sedum*, *Sinocrassula*, and *Umbilicus*) (Kim and Kim, 2020; Zhao et al., 2020; Zhao et al., 2022). This suggests that despite being morphologically and cytologically extremely variable, major lineages of the subfamily Sedoideae have highly conserved plastomes. In the case of the hypervariable regions of plastomes (**Figure 3**), which can be used as barcoding markers, we identified one *ycf1* gene and three intergenic regions (*trnH*–*psbA*, *ndhF*–*rpl32*, and *trnW*–*trnP*), which showed high sequence variation in the two sister lineages *Rhodiola* and *Phedimus* (Zhao et al., 2022). These sets of mutation hotspots in the two sister genera could provide useful phylogenetic information for phylogeographic and population genetic studies at the intraspecific level. *Rhodiola* species, which are mainly adapted to alpine habitats in the Qinghai-Tibet Plateau and the Hengduan Mountains, have been shown to contain three positively selected genes (*rpl16*, *ndhA*, and *ndhH*) and one gene with a faster than average rate of evolution (*psaA*) (Zhao et al., 2020). The products of these genes may have been involved in the adaptive radiation of *Rhodiola* to high altitudes, an environment with low CO_2_ concentrations and high-intensity light. In the subg. *Aizoon* of the *Phedimus* lineage, which was demonstrated to have significant niche divergence from its sister *Rhodiola* (Zhao et al., 2020), we identified different sets of positively selected genes (*ccsA*, *cemA*, *matK*, *ndhC*, *psbJ*, *rpl22*, and *rpoC2*) (**Table 2**). Because *Phedimus* species occur in various wider habitats (e.g., grassy slopes, shrub thickets, meadows, rocky streamsides, sandy cliffs, mountain steppes, stony and gravelly soils in forests, sandy shores, deciduous fields and forests, subalpine meadows, limestone hills, and rocks), these plastomic adaptations are assumed to contribute to the diversification and range expansion of subg. *Aizoon* throughout its geographical range. This also suggests that these genes were most likely selected when the common ancestor of the genus *Phedimus* diverged from its sister lineage, *Rhodiola*. However, it is yet to be determined whether some species of subg. *Phedimus*, which occur at high altitudes (e.g., *P. stevenianus*, *P. spurius*, and *P. obtusifolius*), show any evidence of positive selection for the same genes (*ndhA*, *ndhH*, *rpl16*, and *psaA*) detected in *Rhodiola*. It is also necessary to investigate what drives radiating diversification and adaptation to diverse ecological niches (e.g., Kapralov et al., 2013; Zhang et al., 2021; Izuno et al., 2022).

### 4.2 Origin of Korean endemic *Phedimus* species

One major objective of this study was to determine the origin and evolution of the *Phedimus* species endemic to Korea. This study, based on extensive sampling in eastern Asia, allowed us to gain insights into the origin of *P. takesimensis* on Ulleung Island for the first time. A previous study showed the monophyly of *P. takesimensis* on Ulleung Island without concretely determining the closest continental sister lineage(s) (Seo et al., 2020). The current study further corroborates previous findings that the morphological and genetic variation of *P. takesimensis* on Ulleung Island were accumulated after a single colonization event of a continental ancestral population on the island. However, the ITS tree strongly suggests that monophyletic *P. takesimensis* (96% BS) is embedded within paraphyletic *P. middendorffianus* (**Figure 5** and **Supplementary Figure 1**), whereas the species of *Phedimus* from Russia (*P. aizoon*, *P. litoralis*, and *P. selskianus*) are the closest continental relatives based on the plastome tree (100% BS; **Figure 4**). All accessions of *P. middendorffianus* sister to *P. takesimensis*, on Ulleung Island were from northeastern (Jilin) China. It also commonly occurs from eastern Siberia to the Russian Far East. Therefore, this species is a possible continental progenitor species of *P. takesimensis* on Ulleung Island, suggesting cautiously its origin from northeastern Asia as a geographical source area, based on ITS phylogeny (**Figure 5**). Although this geographical region seems likely to be the origin of *P. takesimensis*, the plastome phylogeny suggests that different sets of species, i.e., *P. aizoon*, *P. litoralis*, and *P. selskianus*, are most likely progenitor species (**Figure 4**). Of these three species, *P. selskianus* has a distinct characteristic of densely grayish pubescent leaves. *Phedimus litoralis* is a glabrous herb with an elongated, creeping, simple rhizome, and strong stems. The last species in this clade was *P. aizoon* from Primorsky Krai. Therefore, based on plastome phylogeny, we cautiously suggest that *P. aizoon*-like species from northeastern Asia (Jilin, China, or the Russian Far East) may also be involved in the origin of *P. takesimensis*. The estimated divergence time of *P. takesimensis* from its continental progenitor species was estimated to be 2.03 Ma (95% HPD, 1.15–3.70 Ma), suggesting its origin soon after the formation of Ulleung Island (ca. 1.8 Ma) (**Figure 6**). Of the nearly 40 endemic species on Ulleung Island, *P. takesimensis* is an example of its geographical origin from the Russian Far East and northeastern China rather than from the Korean Peninsula (*Rubus takesimensis*, Yang et al., 2019; *Campanula takesimana*, Cheong et al., 2020; *Prunus takesimensis*, Cho et al., 2021), southern Korean Peninsula/southern Japan (*Rubus takesimensis*, Yang et al., 2019), and Japan/Sakhalin, Russia (*Scrophularia takesimensis*, Gil et al., 2020). The phylogenetic incongruence between maternally inherited plastome sequences and the multicopy nature of nrDNA ITS sequences further complicate the origin of *P. takesimensis*. Additional studies based on genome-wide SNPs (e.g., Cho et al., 2021) or reduced representation sequencing approaches (e.g., Johnson et al., 2019) would be useful for future phylogenetic studies of subg. *Aizoon* in East Asia.

The taxonomic status of *P. zokuriensis* and its relationship with other congeneric species is contentious. It can be distinguished from congeneric species by its weak and creeping stems. Such phenotypic variation from its congeneric species could be caused by its forest habitats and is well within the range of broadly distributed species, such as *P. kamtschaticus* or *P. aizoon*. Because *P. kamtschaticus* tends to occur on rocky surfaces in shaded forests and sunny forest edges instead of the more open grassland habitats of *P. aizoon*, it is highly plausible that *P. zokuriensis* is conspecific to *P. kamtschaticus* or at least closely related to it. Morphologically, *P. zokuriensis* is more closely related to *P. kamtschaticus* than to *P. aizoon*. In addition, based on extensive morphological analyses, Chung and Kim (1989) argued that *P. zokuriensis* and *P. ellacombeanus* are not infraspecific taxa of *P. kamtschaticus*, and this could be difficult to determine based on the ITS phylogeny owing to weak bootstrap support (<50% BS; **Figure 5**). Plastome phylogeny suggests that *P. zokuriensis* is embedded within the clade of *P. ellacombeanus*, which was sampled from the southern Korean Peninsula (100% BS). This plastome connection between accessions of forest habitat from the central Korean Peninsula and coastal habitat from the southern Korean Peninsula, including isolated islands, was unexpected, owing to their geographical distance and morphological differences. Nevertheless, based on a previous extensive morphological investigation (Chung and Kim, 1989) and its monophyly and close maternal relationship with *P. ellacombeanus* from this study, the species status of *P. zokuriensis* would be reasonable to maintain until different lines of evidence suggest otherwise.

*Phedimus latiovalifolium* is also narrowly endemic to Gangwon-do Province in the northern part of South Korea. It was originally described by Lee (1992) but was later hypothesized based on morphological intermediacy to have a hybrid origin between *P. aizoon* and *P. kamtschaticus* or between *P. aizoon* and *P. ellacombeanus* (Lee, 2000). Yoo and Park (2016), based on morphological and allozyme studies, refuted its hybrid origin and suggested a distinct taxonomic status for *P. latiovalifolium*. Our current study, for the first time, demonstrated the monophyly of *P. latiovalifolium* (94% BS, **Figure 5**) and suggested that it shared its most recent common ancestor with broad-leaved maritime *P. ellacombeanus* sampled from Hachuju and Sochi Island (Jeju and Gyeongsangnam-do Province, respectively) and *P. kamtschaticus* (71% BS). However, the complete plastome tree is less conclusive but shows its relationship with close relatives, suggesting that it shares the most recent common ancestor with species from the Russian Far East (*P. selskianus*, *P. litoralis*, and *P. aizoon*) and Ulleung Island endemic *P. takesimensis* in Korea (92% BS, **Figure 4**). Like *P. zokuriensis*, we suggest that it would be reasonable to maintain the species status of *P. latiovalifolium* because of its morphological and allozyme distinctions (Yoo and Park, 2016) and the strong monophyly demonstrated in this study. Unlike the recent origin of two other Korean endemic species, *P. takesimensis* and *P. zokuriensis*, it was suggested that the split of *P. latiovalifolium* from its sister lineage might have occurred at 10.53 Ma (95% HPD, 4.02–34.35 Ma) during the late Miocene (**Figure 6**).

### 4.3 Species boundary within subg. *Aizoon*: Splitter versus lumper

This study allowed us to broadly assess species relationships within *Phedimus*, thus giving us an opportunity to evaluate species boundaries. Within the genus *Phedimus*, two major lineages are recognized: the first clade includes two species of subg. *Phedimus* (*P. stellatus* and *P. spurius*) and two species of subg. *Aizoon* (*P. yangshanicus* and *P. odontophyllus*), while the second one includes all but two species of subg. *Aizoon* (**Figure 5**). Two species (*P. stellatus* and *P. spurius*) in this clade are diploids (*P. odontophyllus* and *P. yangshanicus* of subg. *Aizoon* are unknown) with a simple descending dysploidy series of *x* = 7 to *x* = 6 to *x* = 5 (Hart et al., 1993; Hart and Bleij, 2003). These two species and other members of subg. *Phedimus* are morphologically distinct and owing to their largely contiguous geographical distribution in Eurasia, a recent origin has been suggested (Hart and Bleij, 2003). Although subg. *Aizoon* is a very distinct taxon based on morphological and cytological characteristics (*x* = 8), its component species are much less clear, except for the hirsute *P. selskianus*. Other taxa are very difficult to separate and can be merged because of the uniformity of their floral characters despite the extreme variation in vegetative characters (Hart and Bleij, 2003). However, Hart and Bleij (2003) followed a less rigorous and much more conservative approach, recognizing approximately 14 species in subg. *Aizoon*; Fröderström (1931) included all but one species (*P. hybridus*), either as a subspecies or synonym in *P. aizoon*. We fully agree that large-scale, comprehensive biosystematics studies of natural populations are required to properly understand morphological and cytological variation (Amano, 1990; Amano and Ohba, 1992; Hart and Bleij, 2003). However, our current study provides some insights into the species boundaries in subg. *Aizoon* from East Asia. We found that the monophyletic *P. hybridus* represents the earliest diverged lineage within this subgenus in the ITS phylogeny. Despite the poorly described nature of this species, *P. hybridus* appears to be a distinct taxon within subg. *Aizoon*. The ITS phylogeny suggested that *P. selskianus*, a very distinct hirsute species, is sister to the clade containing *P. sichotensis* (Russia), *P. kamtschaticus* (Japan), *P. kurilensis* (Russia), *P. middendorffianus* (China), and *P. takesimensis* (Korea) (**Figure 5**). According to the plastome phylogeny (**Figure 4**), it is sister to the clade of *P. litoralis*–*P. aizoon* (Russia). Therefore, if *P. selskianus* can be recognized as a distinct species, other species in this “clade 1,” such as *P. sichotensis*, *P. middendorffianus*, and *P. takesimensis* (Hart and Bleij, 2003), could also maintain cautiously distinct species status until different lines of evidence suggest otherwise. We could also maintain distinct species status for *P. sikokianus* (Japan), *P. zokuriensis* (Korea), and *P. latiovalifolium* (Korea) in “clade 2.” *Phedimus sikokianus* has been suggested to be a key species for understanding the evolution of the whole subg. *Aizoon* because of the low diploid chromosome number and the opposite leaves (Amano and Ohba, 1992; Hart and Bleij, 2003). Its phylogenetic position is unresolved in the ITS tree (**Figure 5**), but in the plastome tree, *P. sikokianus* first diverged within the subg. *Aizoon* (**Figure 4**). The precise phylogenetic position of *P. sikokianus* requires further study based on multiple samples and a robust phylogenetic framework. We were unable to include any members of subg. *Phedimus*, but as in the ITS tree, it is still possible that *P. sikokianus* represents one of the early diverged lineages in subg. *Aizoon*, as supported by cytological evidence (Amano and Ohba, 1992).

Although Chung and Kim (1989) argued for the distinct taxonomic status of *P. ellacombeanus*, it is uncertain whether it should be recognized as a distinct taxon in Korea based on the current study. One accession collected from the type locality in Hakodate (Hokkaido, Japan) was embedded within the *A. aizoon* clade in the ITS phylogeny (**Figure 5**). However, some accessions of *P. ellacombeanus* sampled, including those previously reported by Chung and Kim (1989), were positioned in various lineages within “clade 2.” In the plastome phylogeny (**Figure 4**), the type locality *P. ellacombeanus* was sister to the clade of *P. kamtschaticus*–*P. kurilensis* from Russia, whereas two accessions sampled from Korea were closely related to *P. zokuriensis* and *P. middendorffianus*–*P. kamtschaticus* in Korea. The accessions from Korea occurred on seashores or sunny rock surfaces in forests on islands and tended to have broader spatulate leaves. However, we noticed that the leaf characteristics described in previous reports (Praeger, 1917; Praeger, 1921; Jacobsen, 1960; Clausen, 1975; Evans, 1985; Chung and Kim, 1989) were not observed in mature plants in the current study. As we cultivated *P. ellacombeanus* sampled from Geoje and Hachuja Islands at Sungkyunkwan University, we observed that young emerging stems tend to have opposite to subopposite very broad leaves with two to four crenate margins, which fits the general description of *P. ellacombeanus* by Chung and Kim (1989). In addition, two specimens cited by Chung and Kim (1989) as *P. ellacombeanus* from Geoje Island (SNU, 66782) and Chuja Island (SNU, 66785) are very similar to our collections, especially the young Geoje Island accession. Therefore, this study suggests that *P. ellacombeanus*, previously reported in Korea, may not truly represent *P. ellacombeanus* originally described from the type locality in Hakodate, Japan. Given their phylogenetic positions, *P. ellacombeanus* accessions sampled from Korea could be considered *P. kamtschaticus* sensu Korea (= *P. aizoon* var. *floribundus* sensu Japan). Since *P. ellacombeanus* was described as a new species based on cultivated materials and was originally collected as *P. kamtschaticus* by Maximowicz, it is necessary to investigate its taxonomic distinction from *P. kamtschaticus* based on broad sampling, especially from Hokkaido, northern Japan, and southern Kuriles, Russia.

*Phedimus kurilensis* was described by Voroshilov (1965) from the island of Kunashir and is considered endemic to southern Kuriles. Voroshilov later considered *P. kurilensis* to be a subspecies of *P. sikokianus* based on several characteristics (Ohba, 2002). However, *P. sikokianus* is endemic to the southern part of Japan, Shikoku, and occurs in the mountains (Ohba, 2002). Therefore, the distribution and habitat may not support the subspecies treatment of *P. kurilensis* as *P. sikokianus*. The same species of *P. kurilensis* is thought to occur in the northern part of the island of Hokkaido in Japan, perhaps under different names (personal observation, Marina Koldaeva). The ITS phylogeny in our study suggests that *P. kurilensis* is sister to the clade containing *P. middendorffianus* and *P. takesimensis* (**Figure 5**). In addition, the plastome phylogeny suggests that *P. kurilensis* is sister to *P. kamtschaticus*, which was sampled from Hokkaido in our study (100% BS). In the Flora of Japan (Ohba, 2002), *P. kurilensis* is considered a synonym of *P. kamtschaticus* (Uhl and Moran, 1972). This species is known to occur in Kamchatka, the Kuriles, and Japan. The species description of *P. kamtschaticus* in the Flora of Japan (2002) is quite different from that of Korea, China, and Russia and from that by Hart and Bleij (2003). Although *P. kurilensis* and *P. kamtschaticus* appear to be closely related, it is uncertain whether *P. kurilensis* in the southern Kuriles and *P. kamtschaticus* in Hokkaido are conspecific based on species description and chromosome number. Based on the ITS phylogeny (**Figure 5**), it seems that *P. kurilensis* is distinct from the rest of the *P. kamtschaticus* lineages from Korea and China. Given its close relationship with other species currently recognized (*P. selskianus*, *P. sichotensis*, *P. middendorffianus*, and *P. takesimensis*), *P. kurilensis* could maintain its distinct species status until other lines of evidence indicate otherwise.

*Phedimus litoralis* is endemic to the Ussuriysk floristic region (Hart and Bleij, 2003). We sequenced two additional accessions of *P. litoralis* from Primorsky Krai, and these accessions were closely related to accessions of *P. aizoon*, primarily sampled from Primorsky Krai (Russia) and one accession from Heilongjiang (China) (**Figure 5**). In addition, plastome phylogeny suggested that *P. litoralis* is sister to *P. aizoon* sampled from Russia (100% BS). Therefore, as recently suggested by Hart and Bleij (2003), *P. litoralis* could be conspecific to *P. aizoon* or at least closely related to it. This *P. aizoon* lineage, including *P. litoralis*, appears to be distinct from other lineages of *P. aizoon*. *Phedimus sichotensis*, another Russian Far East endemic in southern Primorsky Krai, is almost indistinguishable from many small forms of the variable *P. kamtschaticus* and was considered a subspecies of *P. middendorffianus* (Gontcharova, 2000). ITS phylogeny showed that *P. sichotensis* was sister to the clade containing primarily *P. kurilensis*–*P. middendorffianus*–*P. takesimensis* (73% BS). Therefore, it is highly unlikely that *P. sichotensis* is related to *P. kamtschaticus*, but it is closely related to *P. middendorffianus*, which requires further confirmation based on a broader sampling.

Lastly, the taxonomic recognition of two widely occurring species, *P. aizoon* and *P. kamtschaticus*, as separate species or infraspecific levels could be problematic given their polyphyletic nature and close relationships with other more distinct species. Some distinct geographical lineages could be recognized for each species, while two species could be intermixed in certain clades, making the species boundaries of the two species difficult (**Figures 4**, **5**). This could be due, in part, to different species descriptions depending on the country. In the Flora of Japan (2001), two infraspecific taxa were recognized for *A. aizoon* (var. *aizoon* and var. *floribundus*) based on the number of flowering stems and leaves: flowering stems fascicled and oblanceolate to lanceolate-ovate to ovate leaves with apically serrated leaf margins for var. *floribundus* versus flowering stems one or two and rhombic-elliptic to elliptic leaves with regularly serrated leaf margins, except near the base for var. *aizoon*. In Korea, these descriptions match those of *P. kamtschaticus* in the former (var. *floribundus*) and *P. aizoon* in the latter (var. *aizoon*). The species description of *P. kamtschaticus* by Hart and Bleij (2003) fits that of *P. kamtschaticus* in Korea and China and that of *P. aizoon* var. *floribundus* in Japan. Therefore, it is possible that plants with taller, one- to two-pronged stems and more uniform characters could be recognized as *P. aizoon*, whereas plants with smaller and more variable characters could be recognized as *P. kamtschaticus* or *P. aizoon* var. *floribundus*. The species description of *P. kamtschaticus* (Fisch. et C.A. Mey) in Japan seems quite different from that in Korea, China, Russia, and Hart and Bleij (2003). It is described as having numerous slender, fascicled flowering stems, 5–10 cm tall; small oblanceolate leaves (1–2 cm long and 0.6–1 cm wide) with irregularly serrate leaf margins; and carpels patent in fruits. It is known to occur in Hokkaido in Japan, southern Kuriles, and Kamchatka (Russia); it has a chromosome number of *n* = 16 (2*n* = 4*x* = 32, tetraploid) (Uhl and Moran, 1972). *P. aizoon* subsp. *aizoon* (= *P. aizoon*) is a highly polyploid species comprising an aneuploid series with 37 different chromosome numbers, ranging from 2*n* = 71 to 2*n* = 124 (2*n* = 12*x* = 96, dodecaploid, most frequent). In contrast, *P. aizoon* var. *floribundus* sensu Japan (=*P. kamtschaticus sensu* Korea, China, and Russia) is tetraploid (2*n* = 32), hexaploid (2*n* = 48), and octoploid (2*n* = 64) (Ohba, 1982; Amano, 1990; Amano and Ohba, 1992). Therefore, it is plausible that *P. kamtschaticus* sensu Japan (*n* = 16) is conspecific to *P. aizoon* var. *floribundus* sensu Japan. Owing to the existence of different ribotypes, we were unable to generate clean ITS sequences for accessions of *P. kamtschaticus* sampled from Hokkaido, Japan. However, the complete plastome sequence strongly suggests that *P. kamtschaticus* from Japan is closely related to *P. kurilensis* (considered a synonym of *P. kamtschaticus* sensu Korea, China, and Russia) and *P. ellacombeanus* (Hokkaido, Japan) and distantly related to any accessions of *P. aizoon* var. *floribundus* from Japan and *P. aizoon* accessions from Korea and Russia (**Figure 5**). This suggests that *P. kamtschaticus* from Hokkaido and southern Kuriles could represent a distinct taxon from *P. kamtschaticus*, as currently recognized in Korea, China, and Japan.

In summary, the following relationships and diversification processes have been proposed based on complete plastome and nrDNA ITS sequences, pending independent confirmation based on genome-wide nuclear data. After the divergence of *P. sikokianus* and *P. hybridus* within the subgenus *Aizoon* during the mid-Oligocene, the *P. aizoon* and *P. kamtschaticus* lineages diverged further. It is hypothesized that certain distinct geographical lineages of each species have become narrowly occurring local endemics: *P. takesimensis*, *P. zokuriensis*, and *P. latiovalifolium* in Korea; *P. litoralis* and *P. sichotensis* in the Russian Far East. It is also highly plausible that species occurring in northern Japan and the Russian Far East (*P. middendorffianus*, *P. sichotensis*, *P. selskianus*, and *P. kurilensis*) shared their most recent common ancestor and contributed to the origin of the insular endemic *P. takesimensis* on Ulleung Island, Korea.

## Data availability statement

The datasets presented in this study can be found in online repositories. The names of the repository/repositories and accession number(s) can be found below: https://www.ncbi.nlm.nih.gov/genbank/, OP344945-OP344959; https://www.ncbi.nlm.nih.gov/genbank/, OP346879-OP346967.

## Author contributions

YK, S-HK, and S-CK designed the experiments and YK, S-HK, M-SC, MK, TI, MM, and S-CK collected the samples. YK, S-HK, and JY performed the experiments and analyzed the data. YK and S-HK drafted the manuscript and MK, TI, MM, and S-CK revised the manuscript. All authors contributed to the article and approved the submitted version.
