# Supplementary material for: Plastome-based backbone phylogeny of East Asian Phedimus (Subgenus Aizoon: Crassulaceae), with special emphasis on Korean endemics
Source: Front Plant Sci. 2023 Mar 14;14:1089165. doi: 10.3389/fpls.2023.1089165 (PMC10043388; doi:10.3389/fpls.2023.1089165)

Supplementary Material

Plastome-based backbone phylogeny of East Asian *Phedimus* (Subgenus *Aizoon*: Crassulaceae), with special emphasis on Korean endemics

Yongsung Kim^1,#^, Seon-Hee Kim^2, #^, JiYoung Yang^3^, Myong-Suk Cho^4^, Marina Koldaeva^5^, Takuro Ito^6^, Masayuki Maki^6^, and Seung-Chul Kim^4,*^

*** Correspondence**: Seung-Chul Kim: sonchus96@skku.edu

**Supplementary Figure 1**. nrDNA ITS sequence-based ML phylogram of genus *Phedimus*.


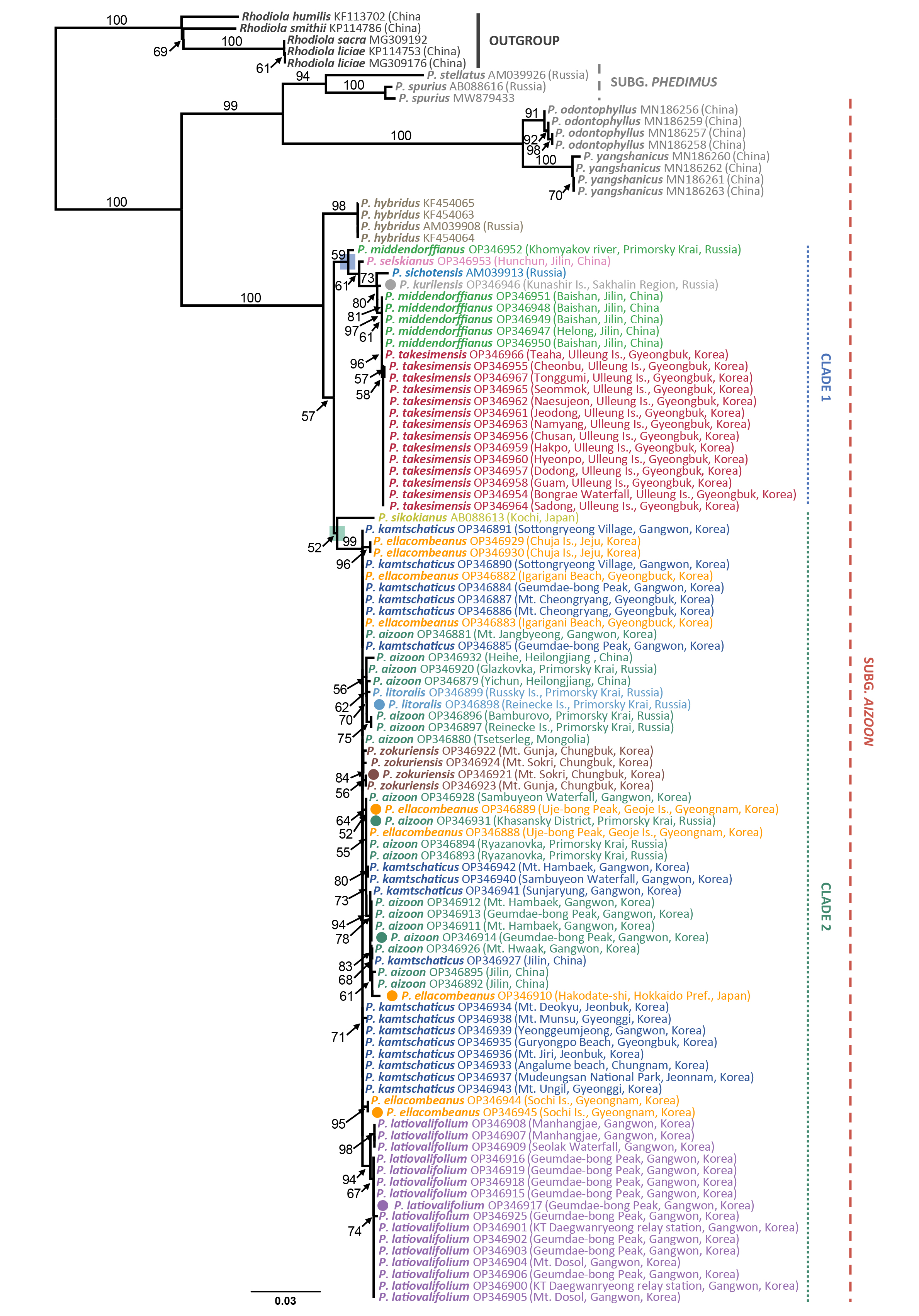

Supplement: Supplementary file 1 [file DataSheet_1.docx]
