# Supplementary material for: Plastome-based backbone phylogeny of East Asian Phedimus (Subgenus Aizoon: Crassulaceae), with special emphasis on Korean endemics
Source: Front Plant Sci. 2023 Mar 14;14:1089165. doi: 10.3389/fpls.2023.1089165 (PMC10043388; doi:10.3389/fpls.2023.1089165)
Supplement: Supplementary file 2 [file Table_1.docx]

Supplementary Material

Plastome-based backbone phylogeny of East Asian *Phedimus* (Subgenus *Aizoon*: Crassulaceae), with special emphasis on Korean endemics

Yongsung Kim^1,#^, Seon-Hee Kim^2, #^, JiYoung Yang^3^, Myong-Suk Cho^4^, Marina Koldaeva^5^, Takuro Ito^6^, Masayuki Maki^6^, and Seung-Chul Kim^4,*^

*** Correspondence**: Seung-Chul Kim: sonchus96@skku.edu

**Supplementary Table 1**. List of species and collection information for *Phedimus* subg. *Aizoon* species used in ITS sequencing. *n.a.*: not available. -: GPS, altitude, voucher not available. (*): obtained from GenBank. Chromosome numbers of *Phedimus* species are from Gontcharova (2006) and ‘t Hart and Bleij (2003).

| **Taxon/ Country** | **Locality** | **GenBank**  **Accession Number** | **GPS** | **Altitude (m)** | **Voucher** |
| --- | --- | --- | --- | --- | --- |
| **1. Genus *Rhodiola* (Outgroup)** | | | | | |
| **(1) *Rhodiola humilis*** | | | | | |
| China | *n.a.* | KF113702 (*) | - | - | - |
| **(2) *Rhodiola liciae*** | | | | | |
| China | *n.a.* | KP114753 (*) | - | - | - |
|  | *n.a.* | MG309176 (*) | - | - | - |
| **(3) *Rhodiola sacra*** | | | | | |
| China | *n.a.* | MG309192 (*) | - | - | - |
| **(4) *Rhodiola smithii*** | | | | | |
| China | *n.a.* | KP114786 (*) | - | - | - |
| **2. Genus *Phedimus*** | | | | | |
| **1) Subgenus. *Aizoon*** | | | | | |
| **(1) *Phedimus aizoon* (chromosome number: 2*n*=56, 80, 71-78, 82, 84, 86, 88, 89, 92-96, 98-113, 116, 124, 128)** | | | | | |
| China | Wudalianchi, Heihe, Heilongjiang | OP346932 | 48 39 5.3 N  126 7 49.7 E | 315 | - |
|  | Wuying District, Yichun, Heilongjiang | OP346879 | 48 7 49.8 N  129 10 54.2 E | 390 | - |
|  | Yanbian Korean Autonomous Prefecture, Jilin | OP346892 | - | - | - |
|  | Yanbian Korean Autonomous Prefecture, Jilin | OP346895 | - | - | - |
| Korea | Geumdae-peak, Jeongseon-gun, Taebaek-si, Gangwon-do | OP34694 | 37°12'15.6"N 128°54'54.6"E | 1,289 | SKK 150804903 |
|  | Geumdae-peak, Jeongseon-gun, Taebaek-si, Gangwon-do | OP346914 | 37°12'15.6"N 128°54'54.6"E | 1,289 | SKK 150804903 |
|  | Mt. Hambaek, Jeongseon-gun, Gangwon-do | OP346911 | 37°09'16.6"N 128°54'53.4"E | 1,334 | - |
|  | Mt. Hambaek, Jeongseon-gun, Gangwon-do | OP346912 | 37°09'16.6"N 128°54'53.4"E | 1,334 | - |
|  | Mt. Hwaak, Sanae-myeon, Hwacheon-gun, Gangwon-do | OP346926 | - | - | - |
|  | Mt. Jangbyeong, Samcheok-si, Gangwon-do | OP346881 | - | - | - |
|  | Sambuyeon Waterfall, Cheorwon-gun, Gangwon-do | OP346928 | 38°08'20.2"N 127°20'05.2"E | 232 | - |
| Mongolia | Tsetserleg | OP346880 | 47 24 45.2 N  102 9 49 E | 1,653 | - |
| Russia | Bamburovo, Primorsky Krai | OP346896 | N42 59.046  E131 18.659 | - | - |
|  | Glazkovka, Primorsky Krai | OP346920 | N43 02.536  E134 10.288 | - | - |
|  | Khasansky District, Primorsky Krai | OP346931 | - | - | - |
|  | Reinecke Island, Vladivostok Urban Okrug, Primorsky Krai | OP346897 | - | - | - |
|  | Ryazanovka, Primorsky Krai | OP346893 | N42 48.489  E131 14.640 | - | - |
|  | Ryazanovka, Primorsky Krai | OP346894 | N42 48.489  E131 14.640 | - | - |
| **(2) *Phedimus ellacombeanus* (chromosome number: unknown)** | | | | | |
| Japan | Hakodate-shi, Hokkaido Pref. | OP346910 | - | - | - |
| Korea | Chuja Islands, Chuja-myeon, Jeju-si, Jeju-do | OP346929 | - | - | - |
|  | Chuja Islands, Chuja-myeon, Jeju-si, Jeju-do | OP346930 | - | - | - |
|  | Igarigani Beach, Pohang-si, Gyeongsangbuck-do | OP346882 | - | - | - |
|  | Igarigani Beach, Pohang-si, Gyeongsangbuck-do | OP346883 | - | - | - |
|  | Sochi Island, Namhae-gun, Gyeongsangnam-do | OP346944 | 34°40'39.2"N 127°56'55.8"E | - | 20170614001 |
|  | Sochi Island, Namhae-gun, Gyeongsangnam-do | OP346945 | 34°40'39.2"N 127°56'55.8"E | - | 20170614001 |
|  | Uje-peak, Nambu-myeon, Geoje-si, Gyeongsangnam-do | OP346888 | - | - | - |
|  | Uje-peak, Nambu-myeon, Geoje-si, Gyeongsangnam-do | OP346889 | - | - | - |
| **(3) *Phedimus hybridus* (chromosome number: unknown)** | | | | | |
| Russia | *n.a.* | AM039908 (*) | - | - | - |
| *n.a.* | *-* | KF454063 (*) | - | - | - |
|  | *-* | KF454064 (*) | - | - | - |
|  | *-* | KF454065 (*) | - | - | - |
| **(4) *Phedimus kamtschaticus* (chromosome number: 2*n*=32, 48, 64)** | | | | | |
| China | Yanbian Korean Autonomous Prefecture, Jilin | OP346927 | - | - | - |
| Korea | Angalume beach, Taean-gun, Chungcheongnam-do | OP346933 | 36°42'19.2"N 126°09'22.8"E | 5 | Seo  SEO0061(SKK) |
|  | Geumdae-peak, Jeongseon-gun, Taebaek-si, Gangwon-do | OP346884 | - | - | - |
|  | Geumdae-peak, Jeongseon-gun, Taebaek-si, Gangwon-do | OP346885 | - | - | - |
|  | Guryongpo Beach, Pohang-si, Gyeongsangbuk-do | OP346935 | 36°01'33.76"N 129°35'10.42"E | 18 | Seo and Yang SEO0068(SKK) |
|  | Mt. Cheongryang, Bonghwa-gun, Gyeongsangbuk-do | OP346886 | - | - | - |
|  | Mt. Cheongryang, Bonghwa-gun, Gyeongsangbuk-do | OP346887 | - | - | - |
|  | Mt. Deokyu, Muju-gun, Jeollabuk-do | OP346934 | 35°52'20.3"N 127°44'37.8"E | 1,253 | Seo and Gil SEO0058(SKK) |
|  | Mt. Hambaek, Jeongseon-gun, Gangwon-do | OP346942 | 37°09'16.6"N 128°54'53.4"E | 1,334 | Kim and Jeon SEO0055(SKK) |
|  | Mt. Jiri, Namwon-si, Jeollabuk-do | OP346936 | 35°17'46.6"N 127°31'46.9"E | 1,428 | Seo and Gil SEO0059(SKK) |
|  | Mt. Munsu, Gimpo-si, Gyeonggi-do | OP346938 | 37°44'21.4"N 126°32'54.7"E | 344 | Seo  SEO0063(SKK) |
|  | Mt. Ungil, Namyangju-si, Gyeonggi-do | OP346943 | 37°34'15.6"N 127°18'07.5"E | 381 | Seo and Gil SEO0053(SKK) |
|  | Mudeungsan National Park Gwangju-si, Jeollanam-do | OP346937 | 35°07'07.6"N 127°59'23.9"E | 75 | Seo and Gil SEO0051(SKK) |
|  | Sambuyeon Waterfall, Cheorwon-gun, Gangwon-do | OP346940 | 38°08'20.2"N 127°20'05.2"E | 232 | Kim and Jeon  SEO0069 (SKK) |
|  | Sottongryeong Village, Goseong-gun, Gangwon-do | OP346890 | - | - | - |
|  | Sottongryeong Village, Goseong-gun, Gangwon-do | OP346891 | - | - | - |
|  | Sunjaryung, Pyeongchang-gun, Gangwon-do | OP346941 | 37°41'23.3"N 128°45'28.1"E | 1,002 | - |
|  | Yeonggeumjeong, Sokcho-si, Gangwon-do | OP346939 | 38°12'46.95"N 128°36'11.86"E | - | Seo  SEO0065(SKK) |
| **(5) *Phedimus kurilensis* (chromosome number: 2*n*=32)** | | | | | |
| Russia | Kosmodemyanskaya Bay, Kunashir Island, Sakhalin Oblast | OP346946 | - | - | - |
| **(6) *Phedimus latiovalifolium* (chromosome number: unknown)** | | | | | |
| Korea | Geumdae-peak, Jeongseon-gun, Taebaek-si, Gangwon-do | OP346915 | 37°12'30.0"N 128°54'56.4"E | 1,337 | SKK150804930 |
|  | Geumdae-peak, Jeongseon-gun, Taebaek-si, Gangwon-do | OP346916 | 37°12'30.0"N 128°54'56.4"E | 1,337 | SKK150804930 |
|  | Geumdae-peak, Jeongseon-gun, Taebaek-si, Gangwon-do | OP346917 | 37°12'30.0"N 128°54'56.4"E | 1,337 | SKK150804930 |
|  | Geumdae-peak, Jeongseon-gun, Taebaek-si, Gangwon-do | OP346918 | 37°12'30.0"N 128°54'56.4"E | 1,337 | SKK150804930 |
|  | Geumdae-peak, Jeongseon-gun, Taebaek-si, Gangwon-do | OP346919 | 37°12'30.0"N 128°54'56.4"E | 1,337 | SKK150804930 |
|  | Geumdae-peak, Jeongseon-gun, Taebaek-si, Gangwon-do | OP346925 | - | - | - |
|  | Geumdae-peak, Jeongseon-gun, Taebaek-si, Gangwon-do | OP346902 | - | - | - |
|  | Geumdae-peak, Jeongseon-gun, Taebaek-si, Gangwon-do | OP346903 | - | - | - |
|  | Geumdae-peak, Jeongseon-gun, Taebaek-si, Gangwon-do | OP346906 | - | - | - |
|  | KT Daegwanryeong relay station, Pyeongchang-gun, Gangwon-do | OP346900 | - | - | - |
|  | KT Daegwanryeong relay station, Pyeongchang-gun, Gangwon-do | OP346901 | - | - | - |
|  | Manhangjae, Gohan-eup, Jeongseon-gun, Gangwon-do | OP346907 | - | - | - |
|  | Manhangjae, Gohan-eup, Jeongseon-gun, Gangwon-do | OP346908 | - | - | - |
|  | Mt. Dosol, Yanggu-eup, Yanggu-gun, Gangwon-do | OP346904 | - | - | - |
|  | Mt. Dosol, Yanggu-eup, Yanggu-gun, Gangwon-do | OP346905 | - | - | - |
|  | Seolak Waterfall, Yangyang-gun, Gangwon-do | OP346909 | - | - | - |
| **(7) *Phedimus litoralis* (chromosome number: 2*n*=64)** | | | | | |
| Russia | Red Stones Bay, Reinecke Island, Vladivostok Urban Okrug, Primorsky Krai | OP346898 | - | - | - |
|  | Vyatlin Peninsula, Russky Island, Vladivostok Urban Okrug, Primorsky Krai | OP346899 | - | - | - |
| **(8) *Phedimus middendorffianus* (chromosome number: 2*n*=64)** | | | | | |
| China | Changbai, Baishan, Jilin | OP346951 | - | - | - |
|  | Helong, Yanbian Korean Autonomous Prefecture, Jilin | OP346947 | 42 1 36.05 N  128 52 53.84 E | 799 | - |
|  | Longgangxiang, Changbai, Baishan, Jilin | OP346948 | 41 32 38.3 N  127 56 44.3 E | 920 | - |
|  | Longgangxiang, Changbai, Baishan, Jilin | OP346949 | 41 32 38.3 N  127 56 44.3 E | 920 | - |
|  | Manjiangzhen, Fusong County, Baishan, Jilin | OP346950 | - | - | - |
| Russia | The environs of Khomyakov river, Pozharsky district, Primorsky Krai | OP346952 | - | - | - |
| **(9) *Phedimus odontophyllus* (chromosome number: unknown)** | | | | | |
| China | *n.a.* | MN186259 (*) | - | - | - |
|  | *n.a.* | MN186256 (*) | - | - | - |
|  | *n.a.* | MN186257 (*) | - | - | - |
|  | *n.a.* | MN186258 (*) | - | - | - |
| **(10) *Phedimus selskianus* (chromosome number: 2*n*=32, 60)** | | | | | |
| China | Hunchun, Yanbian Korean Autonomous Prefecture, Jilin | OP346953 | - | - | - |
| **(11) *Phedimus sichotensis* (chromosome number: 2*n*=32, 48, 64)** | | | | | |
| Russia | *n.a.* | AM039913 (*) | - | - | - |
| **(12) *Phedimus sikokianus* (chromosome number: 2*n*=16)** | | | | | |
| Japan | Kochi Pref. | AB088613 (*) | - | - | - |
| **(13) *Phedimus takesimensis* (chromosome number: unknown)** | | | | | |
| Korea | Bongrae Waterfall, Jeodong-ri, Ulleung-gun, Gyeongsangbuk-do | OP346954 | 37°29'52.6"N 130°53'18.2"E | 296 | Seo  SEO0042(SKK) |
|  | Cheonbu, Cheonbu-ri, Buk-myeon, Ulleung-gun, Gyeongsangbuk-do | OP346955 | 37˚32’20.8"N 130˚52’11.3"E | 18 | Seo  SEO0035(SKK) |
|  | Chusan, Buk-myeon, Ulleung-gun, Gyeongsangbuk-do | OP346956 | 37°32'02.9"N 130°51'07.0"E | 260 | Seo  SEO0039(SKK) |
|  | Dodong, Dodong-ri, Ulleung-gun, Gyeongsangbuk-do | OP346957 | 37°29'05.3"N 130°54'34.9"E | 99 | Seo and Gil SEO0046(SKK) |
|  | Guam, Namseo-ri, Seo-myeon, Ulleung-gun, Gyeongsangbuk-do | OP346958 | 37°28'43.1"N 130°48'32.9"E | 17 | Seo  SEO0031(SKK) |
|  | Hakpo, Seo-myeon, Ulleung-gun, Gyeongsangbuk-do | OP346959 | 37°30'16.9"N 130°48'20.9"E | 51 | Seo  SEO0035(SKK) |
|  | Hyeonpo, Hyeonpo-ri, Buk-myeon, Ulleung-gun, Gyeongsangbuk-do | OP346960 | 37°31'41.2"N 130°49'49.7"E | 5 | Seo  SEO0037(SKK) |
|  | Jeodong, Jeodong-ri, Ulleung-gun, Gyeongsangbuk-do | OP346961 | 37°29'28.6"N 130°54'47.9"E | 14 | Seo and Kim SEO0030(SKK) |
|  | Naesujeon, Jeodong-ri, Ulleung-gun, Gyeongsangbuk-do | OP346962 | 37°30'27.3"N 130°54'33.9"E | 129 | Seo and Kim SEO0001(SKK) |
|  | Namyang, Namseo-ri, Seo-myeon, Ulleung-gun, Gyeongsangbuk-do | OP346963 | 37°28'01.0"N 130°50'11.6"E | 12 | Seo and Kim SEO0012(SKK) |
|  | Sadong, Sadong-ri, Ulleung-gun, Gyeongsangbuk-do | OP346964 | 37°27'31.3"N 130°52'30.6"E | 28 | Seo and Kim  SEO0021(SKK) |
|  | Seommok, Cheonbu-ri, Buk-myeon, Ulleung-gun, Gyeongsangbuk-do | OP346965 | 37°32'33.5"N 130°54'34.6"E | 37 | Seo and Kim SEO0026(SKK) |
|  | Teaha, Teaha-ri, Seo-myeon, Ulleung-gun, Gyeongsangbuk-do | OP346966 | 37˚30’46.2"N 130˚47’53.3"E | 18 | Seo  SEO0033(SKK) |
|  | Tonggumi, Namseo-ri, Seo-myeon, Ulleung-gun, Gyeongsangbuk-do | OP346967 | 37°27'37.3"N 130°51'52.2"E | 34 | Seo and Kim SEO0016(SKK) |
| **(14) *Phedimus yangshanicus* (chromosome number: unknown)** | | | | | |
| China | *n.a.* | MN186260 (*) | - | - | - |
|  | *n.a.* | MN186263 (*) | - | - | - |
|  | *n.a.* | MN186262 (*) | - | - | - |
|  | *n.a.* | MN186261 (*) | - | - | - |
| **(15) *Phedimus zokuriensis* (chromosome number: unknown)** | | | | | |
| Korea | Mt. Gunja, Goesan-gun, Chungcheongbuk-do | OP346922 | - | - | - |
|  | Mt. Gunja, Goesan-gun, Chungcheongbuk-do | OP346923 | - | - | - |
|  | Mt. Sokri, Boeun-gun, Chungcheongbuk-do | OP346921 | - | - | - |
|  | Mt. Sokri, Boeun-gun, Chungcheongbuk-do | OP346924 | - | - | - |
| **2) Subgenus. *Phedimus*** | | | | | |
| **(1) *Phedimus stellatus* (chromosome number: 2*n*=10)** | | | | | |
| Russia | *n.a.* | AM039926 (*) | - | - | - |
| **(2) *Phedimus spurius* (chromosome number: 2*n*=28, 42)** | | | | | |
| Russia | *n.a.* | AB088616 (*) | - | - | - |
| *n.a.* | - | MW879433 (*) | - | - | - |
